# Controlling Helical Asymmetry in Supramolecular Copolymers by *In Situ* Chemical Modification

**DOI:** 10.1021/jacs.3c03411

**Published:** 2023-06-21

**Authors:** Freek V. de Graaf, Stef A. H. Jansen, Tobias Schnitzer, E. W. Meijer, Ghislaine Vantomme

**Affiliations:** † Institute for Complex Molecular Systems, Laboratory of Macromolecular and Organic Chemistry, Eindhoven University of Technology, PO Box 513, 5600 MB Eindhoven, The Netherlands; ‡ School of Chemistry and RNA Institute, University of New South Wales, 2052 Sydney, Australia

## Abstract

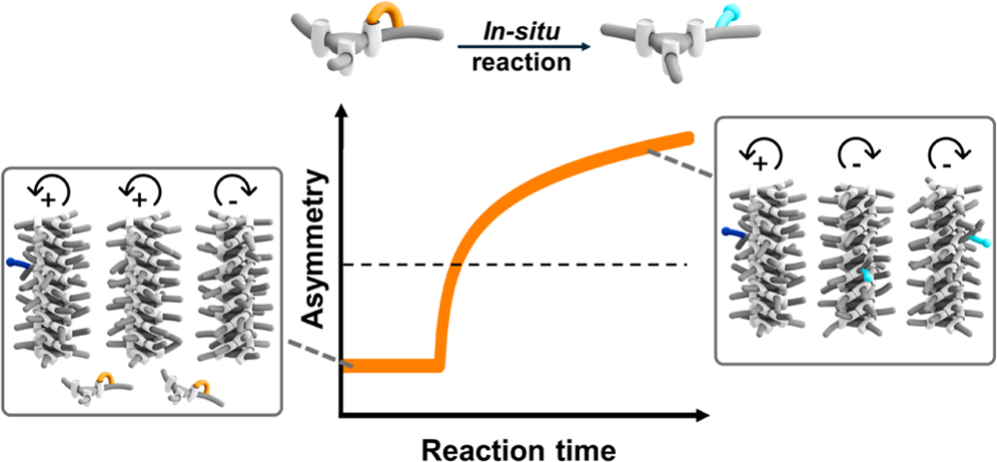

Amplification of asymmetry in complex molecular systems results from a delicate interplay of chiral supramolecular structures and their chemical reactivity. In this work, we show how the helicity of supramolecular assemblies can be controlled by performing a non-stereoselective methylation reaction on comonomers. By methylating chiral glutamic acid side chains in benzene-1,3,5-tricarboxamide (BTA) derivatives to form methyl esters, the assembly properties are modulated. As reacted comonomers, the methyl ester-BTAs induce a stronger bias in the screw-sense of helical fibers predominantly composed of stacked achiral alkyl-BTA monomers. Hence, applying the *in situ* methylation in a system with the glutamic acid-BTA comonomer induces asymmetry amplification. Moreover, mixing small quantities of enantiomers of glutamic acid-BTA and glutamate methyl ester-BTA in the presence of the achiral alkyl-BTAs leads to deracemization and inversion of the helical structures in solution *via* the *in situ* reaction toward a thermodynamic equilibrium. Theoretical modeling suggests that the observed effects are caused by enhanced comonomer interactions after the chemical modification. Our presented methodology enables on-demand control over asymmetry in ordered functional supramolecular materials.

## Introduction

The origin of homochirality in nature is a remarkable and active research topic. Mechanisms that could cause symmetry breaking and asymmetry amplification are widely discussed, yet experimental substantiation is lacking.^1−5^ An exception is the Soai reaction, which shows unprecedented forms of amplification of molecular asymmetry in a chemical system.^6−8^ Asymmetry is widespread in nature *via* the enantioselective use of l-amino acids and d-(deoxy)ribose to produce homochiral superstructures like α-helices and deoxyribonucleic acid (DNA) double helices through supramolecular interactions.^9,10^ The out-of-equilibrium structures derived from these complex systems are maintained by dissipative processes (*i.e.*, energy-consuming covalent reactions) that affect enantioselective noncovalent interactions between macromolecules.^11,12^ Perfect examples are the formation and disintegration of microtubuli during cell proliferation and the recruitment of molecular chaperones to prevent the formation of the thermodynamically favored plaques by amyloid-β precursor proteins.^13,14^ In synthetic chemistry too, numerous reports have applied *in situ* covalent reactions to alter supramolecular assembly properties of monomers, resulting in switchable, adaptive, and even oscillating molecular systems.^12,15−21^ Likewise, *in situ* covalent reactions on the constituents of noncovalent structures can be effective in studying the amplification of asymmetry.^19,22,23^ However, such studies are scarce in literature while these contribute to elucidate the mechanisms leading to homochirality and provide new methodologies to incorporate supramolecular asymmetry in purely synthetic systems.

Supramolecular polymers are of interest in amplification of asymmetry studies due to the ability to form dynamic chiral M or P helices that change handedness in response to external factors.^24−32^ One supramolecular polymer platform consists of benzene-1,3,5-tricarboxamide (BTA), which is widely studied due to its modularity.^33−35^ These discotic molecules form helical supramolecular polymers in apolar solvents through π–π stacking and intermolecular triple hydrogen bonding. A helical bias, *i.e.*, the preference to adopt P or M helicity, is often introduced *via* the intrinsic molecular chirality of the monomers. Thus, supramolecular polymer systems demonstrate how molecular chirality is translated into macroscopic asymmetry. Consequently, the inherent asymmetry of supramolecular polymer systems can be transferred into reaction products *via* asymmetric metal and organocatalysis.^36−44^

Methods to study the amplification of asymmetry in macromolecules were originally developed by Green and co-workers and were later translated to supramolecular polymer systems.^45−51^ The first method is the Sergeant-and-Soldiers experiment, in which the majority of the system consists of achiral monomers that can adopt a preferred M or P helicity by doping the system with chiral monomers. The second method is the Majority-Rules experiment, in which the net helicity is governed by mixtures of the two enantiomers. The principle relies on the mismatch penalty, an intrinsic value that describes the penalty for a monomer to enter a stack of its unfavored helicity. The combination of the two methods is a Diluted Majority-Rules experiment, in which an enantiomeric excess (*ee*) is introduced in the sergeant fraction of a Sergeant-and-Soldiers experiment.^52−54^ The abovementioned experiments result in nonlinear translation of molecular chirality into macroscopic asymmetry and fully rely on thermodynamic equilibria that result from mixing of (a)chiral comonomers. So far, two reports have demonstrated the combination of supramolecular polymers with covalent reactions to amplify the supramolecular asymmetry.^22,23^ Herein, *in situ* reactivity is combined with the Sergeant-and-Soldiers principle to trap the supramolecular chirality into molecular chirality, enhancing the *ee* of supramolecular monomers. As a consequence, the asymmetry of the aggregated species is amplified. We propose that performing the reaction directly on the sergeant instead of the soldier would lead to a more effective and robust amplification of asymmetry on the supramolecular structures in thermodynamic equilibrium.

**Scheme 1:**
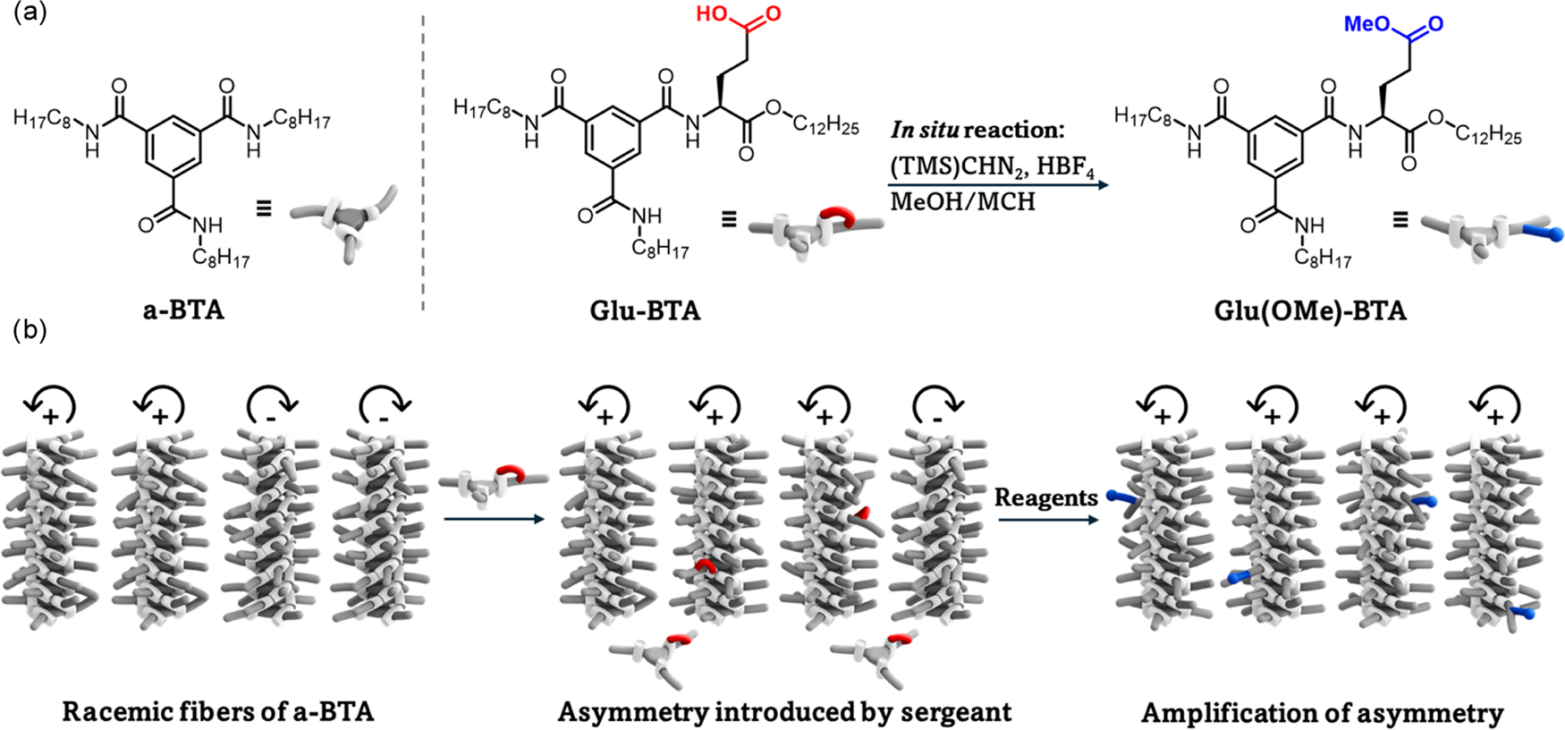
(a) Chemical Structures of BTA Monomers Presented in the Study; *In Situ* Methylation of **Glu-BTA** Yields **Glu(OMe)-BTA** and (b) Schematic Representation of the Amplification Mechanism upon Addition of Reagents to a Solution of Soldiers **a-BTA** and Sergeant **Glu-BTA**

Here, we introduce a noncovalent BTA-based Sergeant-and-Soldiers system comprising chiral sergeants that are functionalized with a glutamic acid residue as a handlebar for *in situ* covalent methylation (Scheme 1a). We demonstrate that the methylation immediately results in the amplification of asymmetry on the helical structures in solution (Scheme 1b), which can be monitored by electronic circular dichroism (CD) spectroscopy. By selectively mixing enantiomers of the sergeant before and after methylation, we can control amplification and inversion of asymmetry, as well as deracemization upon performing the *in situ* reaction.

## Results and Discussion

### Supramolecular Homopolymerization of **Glu-BTA** and **Glu(OMe)-BTA**

We started our investigation by characterizing the individual homoaggregates of our target molecules to highlight differences in their relative stability. **Glu-BTA** was synthesized *via* amidation of an asymmetric monoacid precursor with a dodecyl-extended glutamic acid (Scheme 1a). The optimization, synthetic procedure, and characterization of this specific molecular design are discussed in the supporting information (Figures S1 and S2). We chose the asymmetric design to promote the formation of elongated one-dimensional (1D) structures and decrease the morphological transition from fibers to dimers, which has been demonstrated for symmetric ester-BTAs.^55^ The carboxylic acid moiety in the glutamic acid side chain was expected to interfere with the intermolecular hydrogen bonding and retard supramolecular polymerization, as shown by kinetic evolution experiments (Figure S3a).^56,57^ Moreover, this carboxylic acid was identified as a target for chemical modification, of which the reaction product was expected not to interfere with the intermolecular hydrogen bonding. Thus, we aimed to *in situ* methylate the carboxylic acid to promote supramolecular polymerization and found that (trimethylsilyl)diazomethane ((TMS)CHN_2_) was reactive and selective in the desired assembly conditions (Figure S1). Methylation with (TMS)CHN_2_ yielded the stable product **Glu(OMe)-BTA** (Scheme 1a),^58^ which allowed us to characterize both molecules in their thermodynamically stable state.

**Figure 1:**
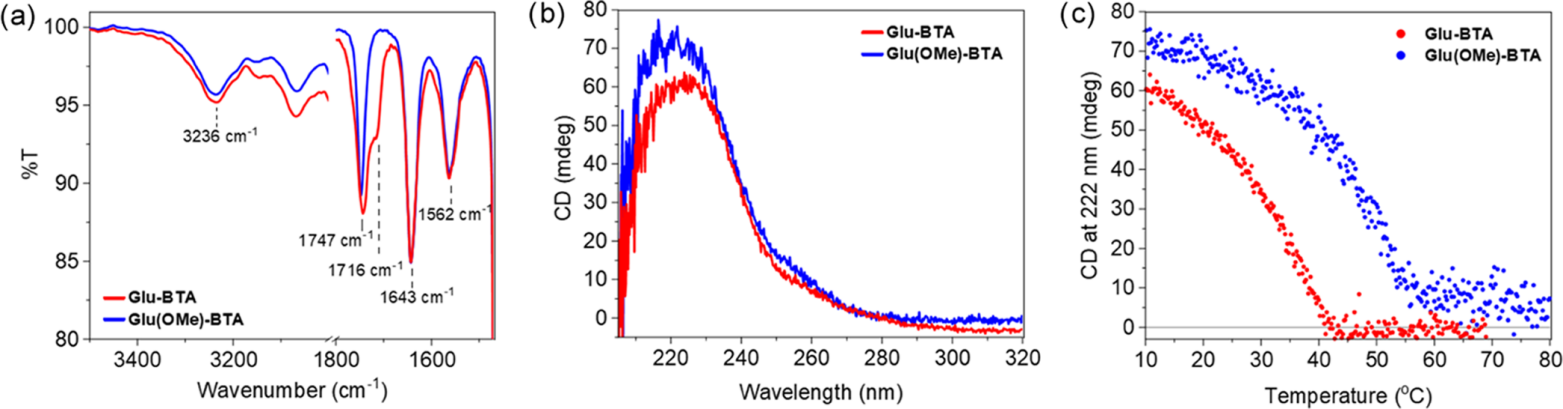
Spectroscopic measurements of **Glu-BTA** (red) and **Glu(OMe)-BTA** (blue) in MCH. (a) FTIR spectra of 1 mM solutions at room temperature. (b) CD spectra of 50 μM solutions at 10 °C. (c) Temperature-dependent CD of 50 μM solutions monitored at 222 nm, cooling rate: 1 K min^–1^.

Homopolymerizations of **Glu-BTA** and **Glu(OMe)-BTA** in methylcyclohexane (MCH) were characterized at room temperature by Fourier transform infrared (FTIR) and CD spectroscopy. The FTIR spectra show absorption bands at 3236 cm^–1^ (bonded N–H stretch), 1643 cm^–1^ (bonded C=O amide I vibrational mode), and 1562 cm^–1^ (bonded C=O amide II vibrational mode) that are typical for threefold hydrogen bonding between amides of the BTA core in supramolecular polymers (Figure 1a).^59^ These absorption bands were not observed for 1 mM samples in acetonitrile (Figure S3b). Furthermore, the band at 1747 cm^–1^ can be ascribed to an unbound ester C=O, indicating no formation of dimeric aggregates.^55^ The shoulder at 1716 cm^–1^ in the spectrum of **Glu-BTA** could be ascribed to bonded carboxylic acid moieties, indicating a bifurcated hydrogen bond of the amide C=O in the aggregated state.^60^

The CD spectra of 50 μM samples of both **Glu-BTA** and **Glu(OMe)-BTA** exhibit Cotton effects with positive maxima at 222 nm (Figure 1b). These spectra are indicative of the presence of single helical BTA fibers with one preferred handedness. The calculated dissymmetry factor (*g-*factor) at room temperature is larger for homopolymers of **Glu(OMe)-BTA** compared to **Glu-BTA** (Figure S5). We observed elongation temperatures (*T*_e_) of 42 and 57 °C for **Glu-BTA** and **Glu(OMe)-BTA**, respectively, by conducting variable-temperature CD measurements (Figure 1c). These measurements suggest that both BTA polymers form *via* a nucleation–elongation cooperative mechanism.^33,61,62^ Thermodynamic analysis was performed to quantify the stability of both homopolymers. The temperature-dependent data was subjected to Van‘t Hoff analysis and fitting procedures using thermodynamic mass-balance models (for details, see the Supporting Information).^63^ The extracted thermodynamic parameters and the resulting binding constants at 20 °C in the elongation phase of homopolymerization (*K*_e_) from these two methods were comparable. The calculated *K*_e_ values of **Glu-BTA** and **Glu(OMe)-BTA** were 2 × 10^5^ and 5 × 10^5^, respectively, confirming that polymers of **Glu(OMe)-BTA** are more stable.

### Copolymerization of Chiral and Achiral BTAs

**Figure 2:**
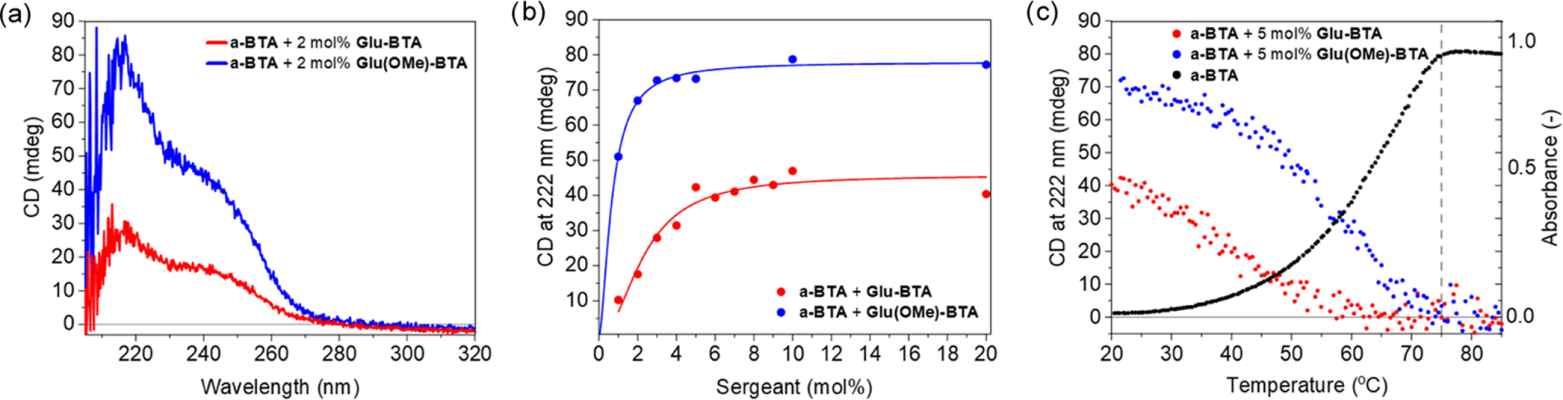
Sergeant-and-Soldiers experiments of sergeants **Glu-BTA** (red) and **Glu(OMe)-BTA** (blue) with soldier **a-BTA** at *c*_tot_ = 50 μM in MCH. (a) CD spectra at 20 °C of samples containing 2 mol % sergeant. (b) CD signal at 222 nm as a function of sergeant fraction at 20 °C. Lines are drawn to guide the eye. (c) Temperature-dependent CD at 222 nm (red and blue) and UV–vis (black) measurements demonstrating the assembly of soldiers and introduction of a helical bias upon cooling at 1 K min^–1^. The *T*_e_ of soldier polymerization at 75 °C is indicated by the dashed line.

We studied the role of **Glu-BTA** and **Glu(OMe)-BTA** in Sergeant-and-Soldiers experiments to explore the potential for asymmetry amplification by *in situ* chemical modification of the sergeant. In separate experiments, the sergeants **Glu-BTA** and **Glu(OMe)-BTA** were mixed with **a-BTA** soldiers in ratios between 1 and 20 mol % (Scheme 1a). The CD profiles of these mixtures show the characteristic shape of Sergeant-and-Soldiers BTA copolymers: a maximum at 217 nm and a shoulder at 245 nm as a result of different packing compared to homopolymers of **Glu-BTA** and **Glu(OMe)-BTA** (Figure 2a).^50^ The intercalation of **Glu-BTA** and **Glu(OMe)-BTA** into polymers of **a-BTA** was further corroborated by FTIR spectroscopy, as both copolymer mixtures exhibited identical spectra of polymeric aggregates (Figure S8a). These spectra did not demonstrate a significant proportion of chain ends, indicating that neither **Glu-BTA** or **Glu(OMe)-BTA** is effective as a chain capper. The CD signal at 222 nm is plotted over the sergeant range for both experiments to demonstrate the net helicity of the thermodynamic equilibrium state at each composition (Figure 2b). The graph clarifies that a larger helical bias is introduced in the screw-sense of the copolymers by sergeant **Glu(OMe)-BTA** compared to **Glu-BTA**. Temperature-dependent CD and ultraviolet–visible (UV–vis) measurements provide mechanistic details on the intercalation of sergeants into stacks of soldiers. In the presence of 5 mol % **Glu(OMe)-BTA**, the helical bias is introduced at a temperature of 73 °C, corresponding to the *T*_e_ of **a-BTA** supramolecular polymerization. Hence, the intercalation of this sergeant occurs simultaneously with supramolecular polymerization of the soldiers (Figure 2c). Intercalation of the sergeant **Glu-BTA** seems to be lagged by 10 °C from the *T*_e_ of the soldiers’ homopolymerization. Apparently, the carboxylic acid moiety of the sergeant **Glu-BTA** disfavors intercalation in soldiers’ stacks in comparison to **Glu(OMe)-BTA**. This was further corroborated by conducting a Sergeant-and-Soldiers experiment at variable temperature with the *tert-*butyl protected precursor BTA as the sergeant (see the Supporting Information, compound **4**). The temperature-dependent data perfectly overlaps with that of **Glu(OMe)-BTA** as the sergeant (Figure S8b), despite the sterically demanding *tert*-butyl moiety.

**Figure 3:**
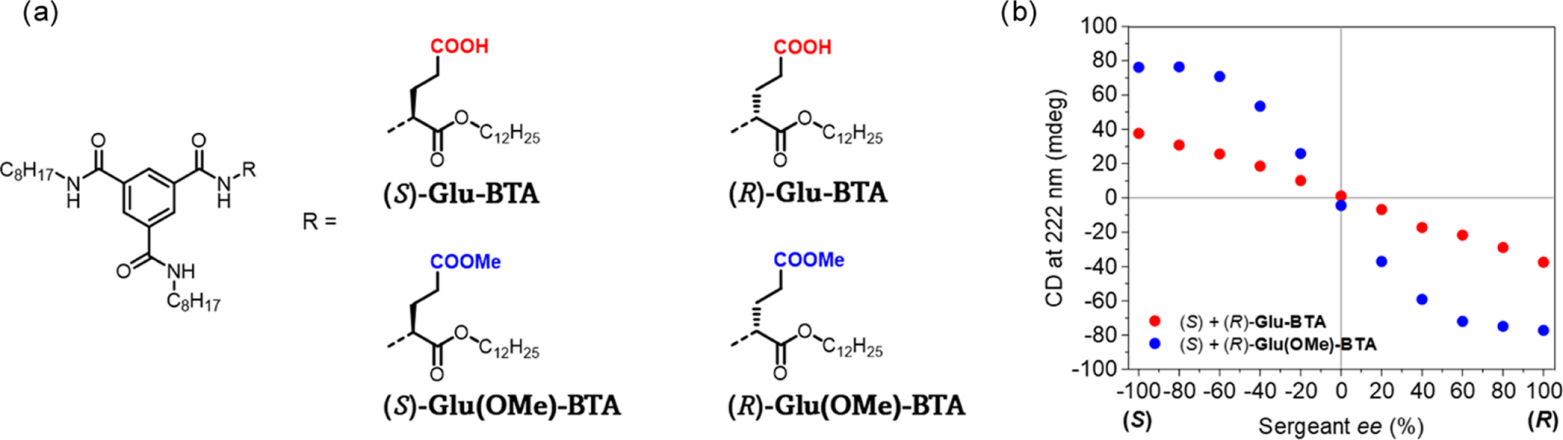
Diluted Majority-Rules experiments. (a) Structures of sergeant enantiomers used in the experiments. (b) Diluted Majority-Rules plot with 5 mol % sergeants (*S*)- and (*R*)-**Glu-BTA** (red) and (*S*)- and (*R*)-**Glu(OMe)-BTA** (blue) with soldier **a-BTA** at *c*_tot_ = 50 μM in MCH, 20 °C.

The differences between **Glu-BTA** and **Glu(OMe)-BTA** as sergeants prompted us to investigate the effect of sergeant *ee* on the asymmetry of copolymers. For this purpose, we conducted Diluted Majority-Rules experiments, where we vary the enantiomeric composition of the sergeant while keeping the sergeant fraction constant at 5 mol % (Figure 3a). The CD at 222 nm is plotted over the range of sergeant *ee* and presented in Figure 3b. For **Glu-BTA**, the relation between *ee* and CD follows a linear trend. This is typical for self-sorting of the two sergeant enantiomers into stacks of their favored helicity, as the enantiomers in minority do not adopt the helicity of the enantiomers in majority.^54^ In contrast, the nonlinear trend resulting from enantiomeric mixtures of **Glu(OMe)-BTA** sergeants indicates a Majority-Rules behavior, *i.e.*, the enantiomers in minority can intercalate into stacks of the unfavored helicity.

### Mechanistic Insights through Simulations of Supramolecular Copolymers

**Figure 4:**
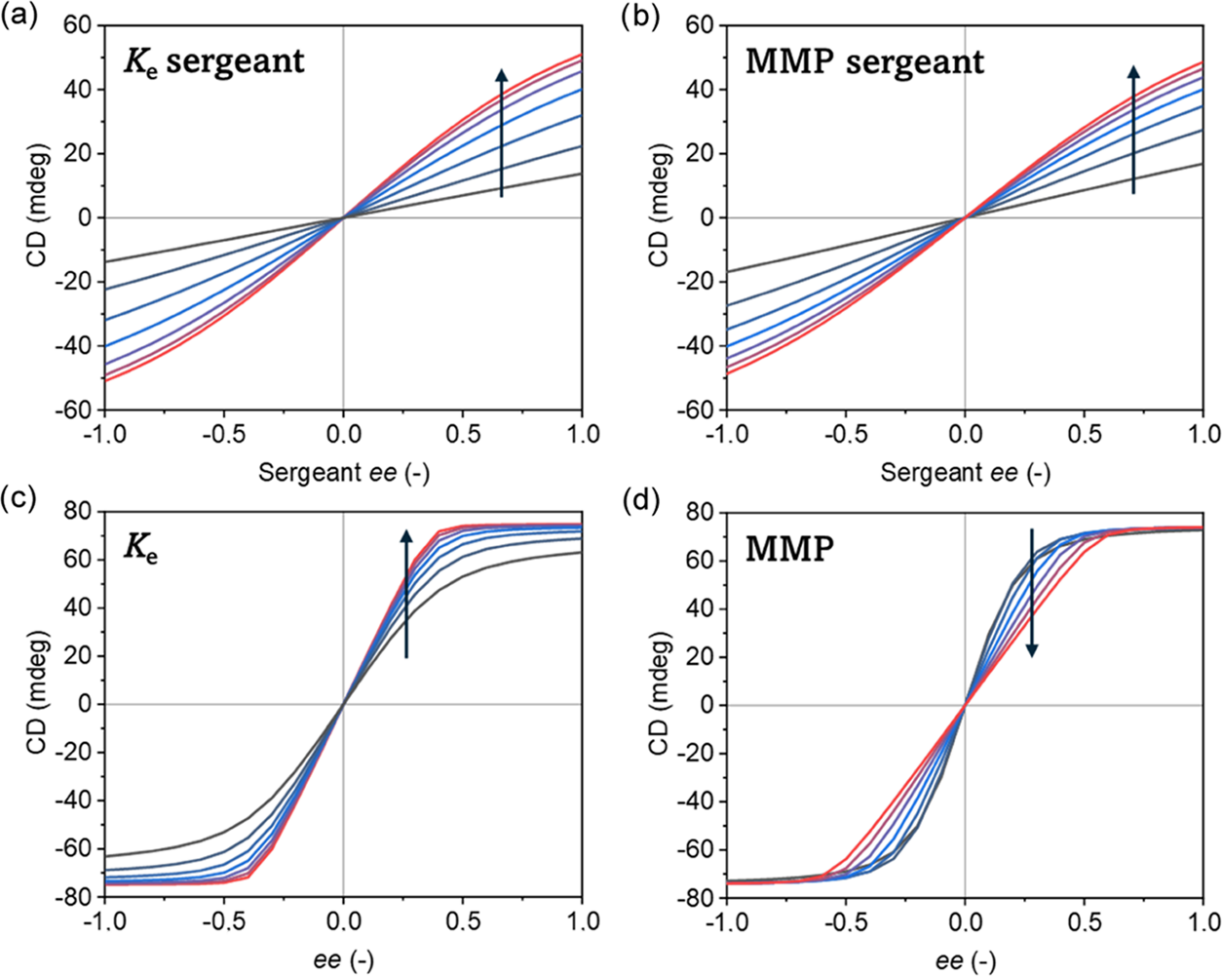
Simulated CD spectra: (a, b) On the effect of variations in sergeant *K*_e_ (with MMP = 1.75 kJ mol^–1^) and MMP (with *K*_e,sergeant_ = 1.02 × 10^5^) on net helicity in a Diluted Majority-Rules experiment. *f*_sergeant_ = 0.05, σ_soldier_ = 0.026, σ_sergeant_ = 0.016, *K*_e,soldier_ = 1.54 × 10^6^. (c, d) On the effect of the monomer *K*_e_ (with MMP = 1.75 kJ mol^–1^) and MMP (with *K*_e_ = 1.02 × 10^5^) in a Majority-Rules experiment. In all figures, the color transition from gray to red represents the increase in the *K*_e_ (range: 1.3 × 10^4^–8.0 × 10^5^) or MMP (range: 0.5–3.0 kJ mol^–1^) value. The arrows are drawn to clarify the effect of the parameter increase on the net helicity.

We used mathematical mass-balance models of two- and three-component copolymerization to simulate the Sergeant-and-Soldiers and Diluted Majority-Rules experiments.^63^ These simulations allowed us to examine the effect of the binding constant (*K*_e_) and mismatch penalty (MMP) of both chiral sergeants **Glu-BTA** and **Glu(OMe)-BTA** on the asymmetry of copolymers. The simulated Sergeant-and-Soldiers (Figure S10) and Diluted Majority-Rules experiments (Figure 4a,b) show that variations in the *K*_e_ and MMP affect the helical bias in a similar manner. For both simulated experiments, an increase in *K*_e_ or MMP gives a stronger asymmetry amplification as recognized by the increased nonlinear response of CD with respect to the sergeant *ee*. Previous reports have shown that BTA-based Diluted Majority-Rules experiments follow Sergeant-and-Soldiers thermodynamic behavior, similar to that indicated by our simulations.^51^ However, these simulations do not provide insights into the distinct contribution of either the sergeant’s *K*_e_ or MMP in asymmetry amplification. In contrast, simulations of regular Majority-Rules experiments, *i.e.*, the copolymerization of two enantiomers, did show an opposing effect on the asymmetry of copolymers between variations in *K*_e_ or MMP (Figure 4c,d). Now, an increase in *K*_e_ also increases the nonlinearity of the asymmetry with respect to *ee*, whereas an increase in MMP reduces this nonlinearity. Thus, stronger intermolecular interactions enhance the amplification of asymmetry while diminished mixing of enantiomers decreases copolymer asymmetry. The simulations suggest that we can decouple the individual contributions of *K*_e_ and MMP by conducting Majority-Rules experiments with the enantiomers of **Glu-BTA** and **Glu(OMe)-BTA** (Figure S11). These experimental results showed a stronger asymmetry amplification for **Glu(OMe)-BTA** compared to **Glu-BTA**, indicating either a higher *K*_e_ or a lower MMP for homopolymerization of **Glu(OMe)-BTA** with respect to **Glu-BTA**. Comparing these findings with the Diluted Majority-Rules experiments revealed that the experimental results are in line with simulations, in which we vary *K*_e_, but in contradiction with simulations, in which we vary MMP. Therefore, the combination of experiments and simulations elucidates that methylation of **Glu-BTA** into **Glu(OMe)-BTA** results in an increase in *K*_e_, *i.e*., stronger binding between sergeant and soldiers. This stronger interaction leads to an enhanced translation of molecular chirality into supramolecular asymmetry in thermodynamically stable copolymers.

### Controlling Asymmetry *viaIn Situ* Methylation

**Figure 5:**
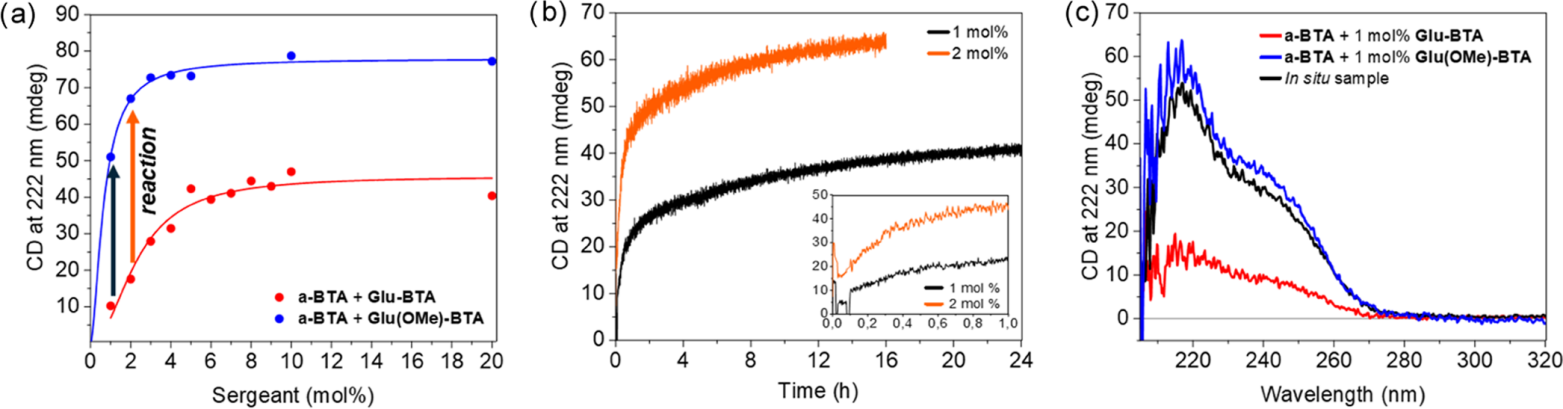
*In situ* methylation of **Glu-BTA** in Sergeant-and-Soldiers experiments at *c*_tot_ = 50 μM, 20 °C in the presence of 70 equiv (TMS)CHN_2_ with respect to the sergeant in MCH. (a) Sergeant-and-Soldiers plots with arrows indicating the potential effect on net helicity by the methylation of 1 mol % (black) and 2 mol % (orange) **Glu-BTA**. (b) CD time course measurements upon addition of reagents in the presence of 1 mol % (black) and 2 mol % (orange) sergeant **Glu-BTA**. (c) Full CD spectra of an *in situ* methylation experiment 4 days after the addition of reagents (black), in comparison with the starting solution containing 1 mol % **Glu-BTA** (red) and a reference solution of 1 mol % **Glu(OMe)-BTA** (blue).

The difference in *K*_e_ for **Glu-BTA** and **Glu(OMe)-BTA** to bind to soldier stacks was exploited for the purpose of on-demand amplification of asymmetry. The *in situ* reaction procedure was optimized as described in the SI (Figures S12–S14). The expected amplification can be expressed as the ΔCD, which is calculated as the difference in CD value at 222 nm between the copolymer systems with **Glu-BTA** versus **Glu(OMe)-BTA** as the sergeant (Figure S9). First, we optimized the *in situ* reaction conditions to be compatible with the conditions for supramolecular polymerization as described in the Supporting Information. Next, applying our optimized reaction procedure to Sergeant-and-Soldiers mixtures of **Glu-BTA** and **a-BTA** copolymers resulted in amplification of asymmetry (black and orange arrows in Figure 5a). The kinetic traces show a strong increase in CD signal within the first hours of the reaction, which gradually slows down over the following reaction time course (Figure 5b). After 16 h, the *in situ* reaction sample starting at 2 mol % **Glu-BTA** reached 96% of the calculated ΔCD. The *in situ* reaction starting with 1 mol % **Glu-BTA** proceeded slower, but the CD measurement after 4 days revealed that 85% of the calculated ΔCD was reached (Figure 5c). Therefore, both *in situ* experiments demonstrate good conversion of **Glu-BTA** into **Glu(OMe)-BTA***via* their distinct effect on the asymmetry of primarily soldier-containing copolymers.

**Figure 6:**
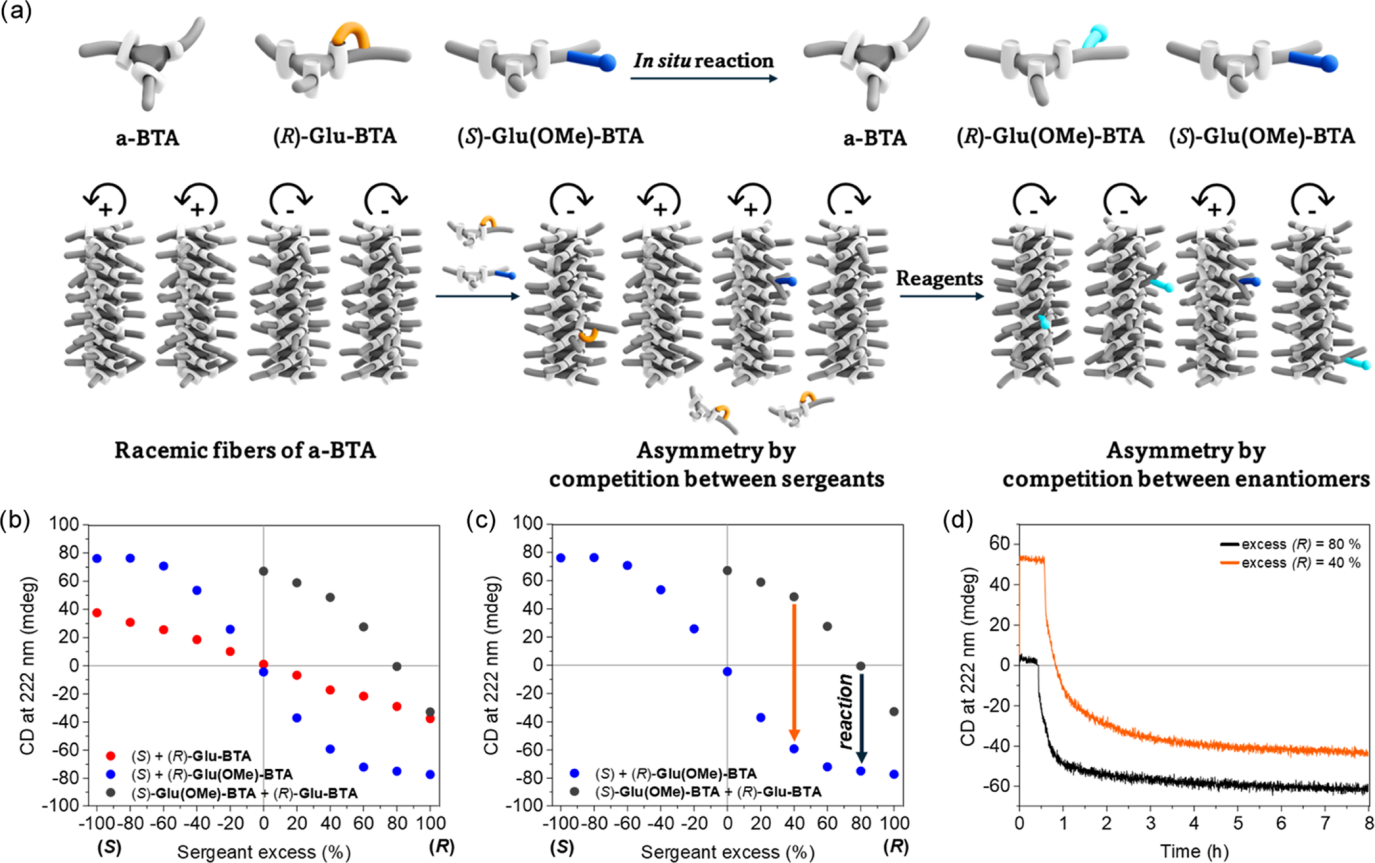
(a) Schematic representation of the *in situ* methylation in sergeant mixtures of (*R*)-**Glu-BTA** and (*S*)-**Glu(OMe)-BTA** with **a-BTA**. (b) Diluted Majority-Rules plot with 5 mol % sergeants (*S*)- and (*R*)-**Glu-BTA** (red), (*S*)- and (*R*)-**Glu(OMe)-BTA** (blue) and mixtures of (*R*)-**Glu-BTA** and (*S*)-**Glu(OMe)-BTA** (gray). (c) Arrows in the plot indicate the effect on net helicity upon methylation of (*R*)-**Glu-BTA** into (*R*)-**Glu(OMe)-BTA**. (d) CD time course measurements upon addition of reagents (after *t* = ±0.5 h) in solutions with 40% (orange) and 80% (black) excess of (*R*)-**Glu-BTA**. (b–d) All experiments were conducted at *c*_tot_ = 50 μM, 20 °C in MCH with 95 mol % **a-BTA**.

Additional intriguing consequences of the *in situ* reaction on the macroscopic asymmetry were realized by mixing enantiopure (*R*)-**Glu-BTA** with (*S*)-**Glu(OMe)-BTA** in a Diluted Majority-Rules system with 95 mol % **a-BTA** (Figure 6a). We changed the ratio of (*R*)-**Glu-BTA /** (*S*)-**Glu(OMe)-BTA** from 1/1 to 1/0 and observed drastically shifted CD values compared to the Diluted Majority-Rules experiments that included only enantiomers of either **Glu-BTA** or **Glu(OMe)-BTA** (Figure 6b). Now, the asymmetry is strongly directed by the (*S*)-**Glu(OMe)-BTA** sergeant even up to 80% excess of (*R*)-**Glu-BTA** sergeant, where a racemic state of the helices in solution is obtained. In these mixtures, the addition of the reagents for *in situ* methylation will selectively convert enantiomers of **Glu-BTA** into **Glu(OMe)-BTA**, as illustrated in Figure 6a. Therefore, the expected transition in the asymmetry (ΔCD) will be toward the measured CD for the Diluted Majority-Rules experiment with enantiomers of **Glu(OMe)-BTA**. Accordingly, the arrows in Figure 6c illustrate two new effects on the asymmetry that could be observed upon *in situ* chemical modification: inverting the asymmetry of the major screw-sense (orange arrow) and deracemization of helices in solution (black arrow). Addition of the reagents in a mixture containing 70 mol % (*R*)-**Glu-BTA** in the sergeant fraction (an excess of 40%) indeed resulted in an inversion of the CD signal. Here, an absolute CD change of 92 mdeg within 8 h was obtained (Figure 6d, orange trace), which is 85% of the calculated ΔCD (Figure S9b). Similarly, the introduction of the reagents in the CD-silent mixture containing 90 mol % (*R*)**-Glu-BTA** in the sergeant fraction (an excess of 80%) resulted in deracemization. Here, an absolute CD change of 62 mdeg was observed within 8 h (Figure 6d, black trace), which is 82% of the calculated ΔCD. Both *in situ* reaction experiments exhibited the largest effect on the asymmetry within the first hour, proving effective and quick on-demand control over the asymmetry of supramolecular helices in solution.

## Conclusions

We have demonstrated that covalent and noncovalent chemistry can be combined to precisely control the asymmetry in supramolecular polymers under thermodynamic equilibrium. When mixed with achiral comonomers, the covalent methylation of the carboxylic acid side chain of chiral monomers enhanced the intermolecular interactions and thus participation in supramolecular polymerization. The observed macroscopic effect is a stronger helical bias upon chemical conversion of the chiral monomers. Variations in the enantiomeric composition of the chiral comonomer fraction enabled controlled effects on the asymmetry, which are initiation (*i.e.*, deracemization), amplification, and inversion of asymmetry by the addition of chemical reagents.

The presented work is an illustration of how a multistep approach of covalent and noncovalent reaction steps *in situ* can be employed as a mean to control asymmetric features in molecular systems. We propose that such simple systems can greatly contribute to our understanding of mechanisms toward homochirality in complex systems.

## Supporting Information Available

Synthetic procedures; materials and methods; additional FTIR, UV–vis, CD spectra; details on thermodynamical analysis and simulations; and details on *in situ* reaction procedures (PDF)
