# Supplementary material for: Controlling Helical Asymmetry in Supramolecular Copolymers by In Situ Chemical Modification
Source: J Am Chem Soc. 2023 Jun 21;145(26):14379–86. doi: 10.1021/jacs.3c03411 (PMC10326880; doi:10.1021/jacs.3c03411)
Supplement: Supplementary file 1 — ja3c03411_si_001.pdf [file ja3c03411_si_001.pdf]

Supporting Information for:

Controlling Helical Asymmetry in Supramolecular Copolymers by *In Situ* Chemical Modification

Freek V. de Graaf[1], Stef A. H. Jansen[1], Tobias Schnitzer[1], E. W. Meijer[1,2] and Ghislaine Vantomme[1]**

[1]Institute for Complex Molecular Systems, Laboratory of Macromolecular and Organic Chemistry, Eindhoven University of Technology, PO Box 513, 5600 MB, Eindhoven, The Netherlands. [2]School of Chemistry and RNA Institute, University of New South Wales, 2052 Sydney, Australia.

Table of Contents

MATERIALS AND METHODS **S2**

SYNTHETIC PROCEDURES **S3**

COMPUTATIONAL ANALYSIS OF ONE-, TWO- AND THREE-COMPONENT SUPRAMOLECULAR POLYMERIZATIONS **S7**

OPTIMIZATION OF GLU-BTA MOLECULAR DESIGN **S10**

ADDITIONAL FIGURES ON THE CHARACTERIZATION OF SUPRAMOLECULAR HOMOPOLYMERS **S12**

THERMODYNAMIC ANALYSIS OF HOMOPOLYMERS **S13**

ADDITIONAL FIGURES ON THE CHARACTERIZATION OF SUPRAMOLECULAR COPOLYMERS **S16**

SERGEANT-AND-SOLDIERS SIMULATIONS **S17**

MAJORITY-RULES EXPERIMENTS **S17**

OPTIMIZATION OF THE *IN SITU* METHYLATION PROCEDURE **S18**

^{1}H NMRS **S21**

^{13}C NMRS **S24**

MALDI-TOF MASS SPECTRA **S26**

REFERENCES **S28**

Materials and Methods

Z-L-Glu(OtBu)-OH and Z-D-Glu(OtBu)-OH were purchased from Chem-Impex Int'l Inc. The purity of these compounds as stated by the supplier are > 98% (HPLC) with chiral purity of > 99.5%. Dodecanol and (trimethylsilyl)diazomethane 2M in hexane ((TMS)CHN_2) were purchased from Acros Organics. EDC·HCl was purchased from TCI Europe. All other chemicals were purchased from Sigma-Aldrich, including spectroscopic grade methylcyclohexane (MCH). MCH and chloroform were dried over molsieves before use in synthesis or spectroscopic studies. MCH was stored under nitrogen. Solvents used for synthesis were purchased from Biosolve B.V., deuterated solvents were purchased from Cambridge Isotopes Laboratories. All commercial compounds were used as purchased without further purification. Synthesis of BTA precursor **3** and **a-BTA** are reported elsewhere.[1]

Flash chromatography was performed on a Biotage model Isolera™ Prime flash system, using Biotage Sfär Silica cartridges (particle size 60 μM). Nuclear magnetic resonance (NMR) spectra were recorded on a Bruker 400 MHz Ultrashield spectrometer (400 MHz for ^{1}H NMR; 100 MHz for ^{13}C NMR). Chemical shifts (δ) are expressed in ppm and are referred to the residual peak of the solvent. Multiplicity is abbreviated as s: singlet, d: doublet, t: triple, q: quadruplet or m: multiplet. Matrix assisted laser absorption/ionization-time of flight (MALDI-TOF) mass spectra were obtained on a Bruker Autoflex Speed with α-cyano-4-hydroxycinnamic acid (CHCA) or trans-2-[3-(4-tert-butylphenyl)-2-methyl-2-propenyl-idene]-malononitrile (DCBT) as matrices. Fourier transform-infrared (FTIR) spectra were recorded on a PerkinElmer Spectrum Two spectrometer using a slide holder module and held in a liquid cell equipped with windows of calcium fluoride. Circular dichroism (CD) and UV/vis spectroscopy experiments were performed on a JASCO J-815 spectropolarimeter equipped with either a JASCO Peltier MPTC-490S temperature controller or a JASCO Peltier PFD-425S/15. For all spectroscopic measurements, Hellma Quartz Suprasil cuvettes with an optical pathlength of 1 cm or 0.1 cm were used.

In situ methylation procedure and sample preparation

Experiments were conducted at room temperature. Stock solutions of **Glu-BTA**, **Glu(OMe)-BTA** and **a-BTA** were prepared by weighing in the solid materials, followed by addition of MCH to get a stock solution of 200 μM for **Glu-BTA** and **Glu(OMe)-BTA** and 1 mM for **a-BTA**. The stock solutions were placed in a sonication bath for 10 minutes, subsequently heated to 80 °C and cooled down to room temperature to get homogeneous solutions. Stock solutions of reagents in MCH were freshly prepared. The 1 vol% methanol stock was prepared by adding 10 μL methanol to 0.99 mL MCH. The 0.001 M stock of tetrafluoroboric acid (HBF_4) was prepared by adding 3 μL of HBF_4 diethyl ether complex to 1.997 mL of MCH, followed by vortexing and 15 minutes of sonication to obtain a homogeneous suspension. From this suspension, 10 μL was transferred into a new vial and diluted with 90 μL MCH. The 0.02 M stock solution of (TMS)CHN_2 was obtained by adding 10 μL of 2 M (TMS)CHN_2 in hexane to 0.99 mL MCH. In a 1 cm pathlength cuvette, the BTA stock solutions were mixed with MCH to obtain a final concentration of 50 μM after addition of methanol. The cuvette was placed in the sample holder and the spectroscopic time course measurement was started. The cuvette was then shortly removed from the sample holder to add 90 μL of methanol stock,

4 µL of HBF_4 stock and 10 µL of (TMS)CHN_2 stock. The cuvette was shaken and placed back in the sample holder to monitor the CD signal over time as the reaction proceeded.

Synthetic procedures

Synthesis of amine (S)-2

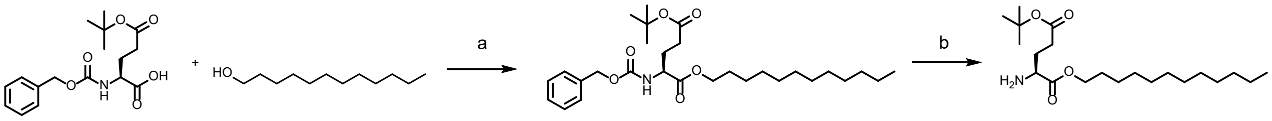

1 **2**

Scheme S1: Synthesis of amine **2**. a) EDC·HCl, DIPEA, THF, 50 °C, overnight, 43 %. b) Pd/C, H_2 atmosphere, MeOH, RT, 5 h, 98 %.

Z-L-Glu(OtBu)-OH (1.00 g, 2.96 mmol) and dodecanol (0.665 g, 3.55 mmol) were dissolved in THF (25 mL) and cooled in an ice bath. EDC·HCl (0.625 g, 3.26 mmol) and DIPEA (0.57 mL, 3.28 mmol) were added, and the ice bath was removed. The reaction was stirred overnight at 50 °C. The THF was removed in vacuo and the sample was dissolved in 50 mL ethyl acetate and washed with water (3 x 20 mL), saturated $NaHCO_3$ (3 x 20 mL) and brine and dried with Na_2SO_4. The organic fraction was concentrated and purified with flash chromatography using a solvent gradient of heptane/ethyl acetate from 9/1 to 3/1 v/v. The resulting fractions were partially contaminated with unreacted dodecanol, only pure fractions were used in the following deprotection step. The pure fractions were concentrated in vacuo and dissolved in methanol (10 mL). Pd/C (10 % w/w, 100 mg) was added, and the mixture was stirred under hydrogen atmosphere at room temperature for 5 hours. The reaction mixture was filtered over a plug of celite and washed with methanol (3 x 5 mL). The solvent was removed in vacuo to give (*S*)-**2** as a slightly yellow waxy solid (0.48 g, 42 %). ^{1}H NMR (400 MHz, Methanol-d_4) δ [ppm] = 4.26 (t, *J* = 6.6 Hz, 2H), 4.10 (t, *J* = 6.6 Hz, 1H), 2.54 – 2.43 (m, 2H), 2.14 (q, *J* = 8.8, 2H), 1.75 – 1.67 (m, 2H), 1.46 (s, 9H), 1.37 – 1.23 (m, 18H), 0.88 (t, *J* = 6.6 Hz, 3H), 13.05. ^{13}C NMR (100 MHz, Methanol-d_4) δ [ppm] = 171.39, 168.92, 80.92, 61.61, 51.81, 32.28, 31.68, 30.14, 29.60 – 28.80, 26.95, 25.54, 22.34, 13.05.

Synthesis of amine (R)-2

An identical procedure as for the synthesis of (*S*)-**2** was followed using Z-D-Glu(OtBu)-OH (1.00 g, 2.96 mmol), dodecanol (0.665 g, 3.55 mmol), EDC·HCl (0.625 g, 3.26 mmol) and DIPEA (0.57 mL, 3.28 mmol). The product (*R*)-**2** was dried under rotary evaporation to yield a slightly yellow waxy solid (0.50 g, 46 %). ^{1}H NMR (400 MHz, Chloroform-*d*) δ [ppm] = 4.11 (t, *J* = 6.7 Hz, 2H), 3.48 (t, *J* = 5.4 Hz, 1H), 2.37 (t, *J* = 7.5 Hz, 2H), 2.08 – 2.00 (m, 1H), 1.93 – 1.81 (m, 1H), 1.64 (p, *J* = 7.1 Hz, 2H), 1.44 (s, 9H), 1.37 – 1.20 (m, 18H), 0.88 (t, *J* = 6.6 Hz, 3H). ^{13}C NMR (100 MHz, Methanol-d_4) δ [ppm] = 172.29, 168.64, 80.46, 61.61, 53.93, 32.28, 31.68, 30.42, 29.46 – 29.33, 26.94, 25.56, 22.34, 13.04.

Synthesis of (S)-4

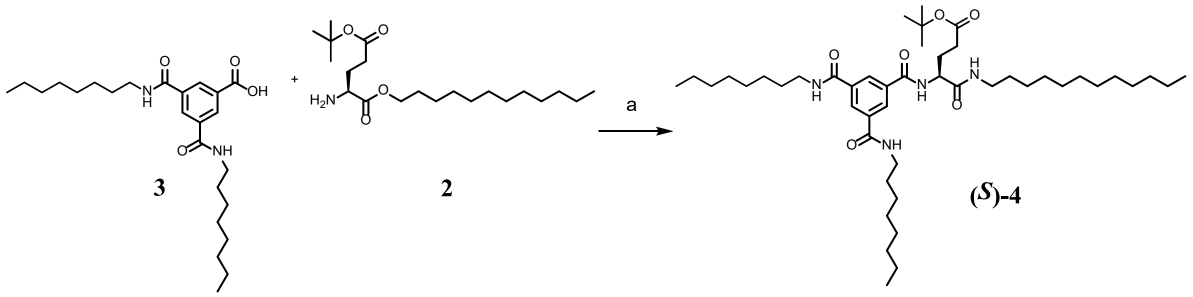

Scheme S2: Synthesis of (*S*)-**4**. a) EDC·HCl, DIPEA, DMAP, HOBt (cat.), dry $CHCl_3$, RT, overnight, 68 %.

In an oven-dried round bottom flask equipped with magnetic stirrer, compound **3** (0.20 g, 0.46 mmol), *(S)*-**2** (0.23 g, 0.6 mmol), DMAP (0.056 g, 0.46 mmol), and HOBt (0.0062 g, 0.046mmol) were dissolved in 15 mL dried chloroform. The mixture was placed under argon and in an ice bath. EDC·HCl (0.13 g, 0.68 mmol) and DIPEA (0.17 mL, 0.92 mmol) were dissolved in 5 mL dry $CHCl_3$ and added through a syringe. The ice bath was removed, and the reaction mixture was stirred overnight at room temperature. The mixture was diluted with 50 mL chloroform and washed with water (3 x 20 mL), 1 M HCl (3 x 20 mL) and saturated $NaHCO_3$ (3 x 20 mL). The organic phase was dried over Na_2SO_4 and concentrated in vacuo on celite. The crude was purified with flash chromatography using a solvent mixture of heptane/ethyl acetate 9/1 v/v. Center fractions of the eluted product were combined and concentrated in vacuo, followed by drying in vacuum oven to give (*S*)-**4** as a slightly yellow solid material (0.248 g, 68 %). ^1H NMR (400 MHz, Chloroform-*d*) δ [ppm] = 8.24 (s, 3H), 7.69 (d, *J* = 7.5 Hz, 1H), 6.68 (t, *J* = 5.7 Hz, 2H), 4.75 – 4.67 (m, 1H), 4.16 (t, *J* = 6.8 Hz, 2H), 3.40 (q, *J* = 7.4, 5.8 Hz, 4H), 2.52 – 2.38 (m, 2H), 2.29 – 2.21 (m, 1H), 2.14 – 2.08 (m, 1H), 1.67 – 1.58 (m, 6H), 1.44 (s, 9H), 1.42 – 1.18 (m, 38H), 0.88 (t, *J* = 6.7 Hz, 9H). ^{13}C NMR (100 MHz, Chloroform-*d*) δ [ppm] = 172.33, 172.27, 166.00, 165.93, 135.61, 134.22, 128.56, 127.91, 81.08, 66.02, 52.79, 40.41, 31.92, 31.83, 31.75, 29.65 – 29.52, 29.34, 29.23, 28.54, 28.08, 27.06, 26.88, 25.86, 22.65, 14.12. MALDI-TOF mass spectrometry: *m/z* ratio calculated for $[C_{46}H_{79}N_3O_7]$+([M·H]+): 786.59 ([M·H]+), observed: 808.58 ([M·Na]+), 824.89 ([M·K]+).

Synthesis of (R)-4

An identical procedure as for the synthesis of (*S*)-**4** was followed using compound **3** (0.257g, 0.59 mmol), (*S*)-**2** (0.34 g, 0.92 mmol), DMAP (0.072 g, 0.59 mmol), HOBt (0.0080 g, 0.059 mmol), EDC·HCl (0.20 g, 1.04 mmol) and DIPEA (0.27 mL, 1.56 mmol). Some contamination was found after the column chromatography and only pure product fractions were combined. The pure product (*R*)-**4** was dried in a vacuum oven to give a slightly yellow solid material (0.187 g, 40 %). ^1H NMR (400 MHz, Chloroform-*d*) δ [ppm] = 8.28 (s, 3H), 7.55 (d, *J* = 7.5 Hz, 1H), 6.57 (t, *J* = 5.7 Hz, 2H), 4.77 – 4.71 (m, 1H), 4.16 (t, *J* = 6.8 Hz, 2H), 3.44 (q, *J* = 7.4, 5.8 Hz, 4H), 2.51 – 2.32 (m, 2H), 2.30 – 2.20 (m, 1H), 2.15 – 2.05 (m, 1H), 1.72 – 1.55 (m, 6H), 1.43 (s, 9H), 1.40 – 1.23 (m, 38H), 0.88 (t, *J* = 6.7 Hz, 9H). ^{13}C NMR (100 MHz, Chloroform-*d*) δ [ppm] = 172.33, 172.27, 166.00, 165.93, 135.61, 134.22, 128.56, 127.91, 81.08, 66.02, 52.79, 40.41, 31.92, 31.83,

31.75, 29.65 – 29.52, 29.34, 29.23, 28.54, 28.08, 27.06, 26.88, 25.86, 22.65, 14.12. MALDI-TOF mass spectrometry: *m/z* ratio calculated for [$C_{46}H_{79}N_3O_7$]+([M·H]+): 786.59 ([M·H]+), observed: 808.58 ([M·Na]+), 824.89 ([M·K]+).

Synthesis of (S)-Glu-BTA

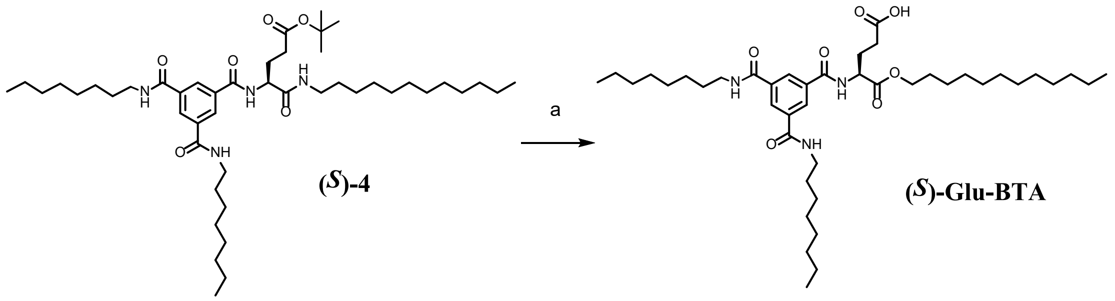

Scheme S3: Synthesis of (*S*)-**Glu-BTA**. a) H_2O, triisopropylsilane, TFA, DCM, RT, 2h, 78 %.

Compound (*S*)-**4** (0.060 g, 0.076 mmol) was dissolved in 3 mL dichloromethane. 2 drops of water and 5 drops of triisopropylsilane were added, followed by 1 mL of trifluoroacetic acid. The mixture was stirred for 2 hours and diluted with 10 mL dichloromethane, washed with saturated $NaHCO_3$ (2 x 5 mL), dried with Na_2SO_4 and concentrated in vacuo. The mixture was dissolved in a small amount of heptane/ethyl acetate 1/1 v/v and purified over a silica plug. The solvent was removed in vacuo and product (*S*)-**Glu-BTA** was dried in vacuum oven to yield a white solid material (0.043 g, 78 %). ^{1}H NMR (399 MHz, Chloroform-*d*) δ [ppm] = 8.34 (d, *J* = 1.6 Hz, 2H), 8.21 (d, *J* = 1.7 Hz, 1H), 8.00 (d, *J* = 7.3 Hz, 1H), 6.97 (t, *J* = 5.7 Hz, 2H), 4.69 – 4.63 (m, 1H), 4.15 (t, *J* = 6.9 Hz, 2H), 3.42 (q, *J* = 7.4, 5.8 Hz, 4H), 2.54 (t, *J* = 7.3 Hz, 2H), 2.42 – 2.16 (m, 2H), 1.69 – 1.59 (m, 6H), 1.43 – 1.22 (m, 38H), 0.87 (t, *J* = 6.7 Hz, 9H). ^{13}C NMR (100 MHz, Chloroform-*d*) δ [ppm] = 175.90, 171.89, 166.33, 165.86, 134.67, 134.00, 128.66, 128.49, 65.91, 53.20, 40.57, 31.92, 31.83, 30.92, 29.65, 29.60, 29.52, 29.36, 29.31, 29.25, 29.23, 28.54, 27.05, 26.46, 25.84, 22.69, 22.65, 14.12, 14.09. MALDI-TOF mass spectrometry: *m/z* ratio calculated for [$C_{42}H_{71}N_3O_7$]+([M·H]+): 730.53 ([M·H]+), observed: 730.55 ([M·H]+), 752.51 ([M·Na]+), 768.47 ([M·K]+).

Synthesis of (R)-Glu-BTA

An identical procedure as for the synthesis of (*S*)-**Glu-BTA** was followed using (*R*)-**4** (0.045 mg, 0.057 mmol). The crude product was purified with flash chromatography using a solvent mixture of heptane/ethyl acetate 1/1 v/v. The solvent was removed and the product (*R*)-**Glu-BTA** was dried in vacuum oven (0.027 g, 65 %). ^{1}H NMR (400 MHz, Chloroform-*d*) δ [ppm] = 8.35 (s, 2H), 8.22 (s, 1H), 7.99 (d, *J* = 7.4 Hz, 1H), 6.95 (t, *J* = 5.7 Hz, 2H), 4.71 – 4.66 (m, 1H), 4.16 (t, *J* = 6.8 Hz, 2H), 3.43 (q, *J* = 7.4, 5.8 Hz, 4H), 2.55 (t, *J* = 7.2 Hz, 2H), 2.39 – 2.20 (m, 2H), 1.64 – 1.60 (m, 6H), 1.40 – 1.23 (m, 38H), 0.88 (t, *J* = 6.9, 9H). ^{13}C NMR (100 MHz, Chloroform-*d*) δ [ppm] = 175.90, 171.89, 166.33, 165.86, 134.67, 134.00, 128.66, 128.49, 65.91, 53.20, 40.57, 31.92, 31.83, 30.92, 29.65, 29.60, 29.52, 29.36, 29.31, 29.25, 29.23, 28.54, 27.05, 26.46, 25.84, 22.69,

22.65, 14.12, 14.09. MALDI-TOF mass spectrometry: *m/z* ratio calculated for $[C_{42}H_{71}N_3O_7]$+([M·H]+): 730.53 ([M·H]+), observed: 730.55 ([M·H]+), 752.51 ([M·Na]+), 768.47 ([M·K]+).

Synthesis of (S)-Glu(OMe)-BTA

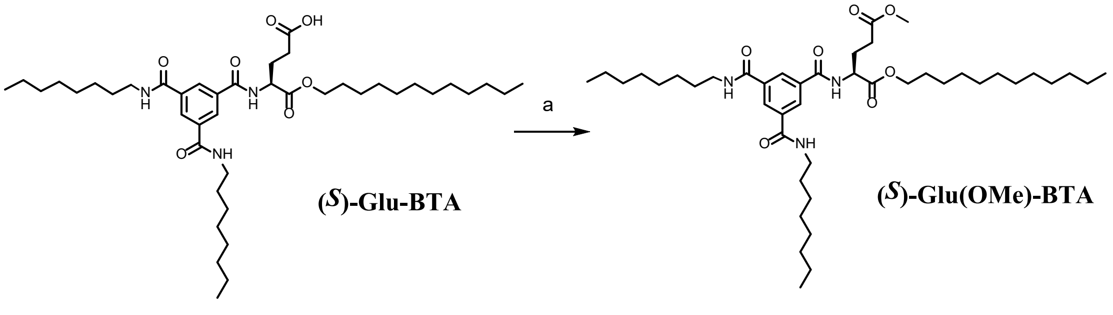

Scheme S4: Synthesis of (*S*)-**Glu(OMe)-BTA**. a) (TMS)CHN_2, toluene/MeOH, RT, 1h, 79 %.

(*S*)-**Glu-BTA** (0.030 g, 0.041 mmol) was dissolved in toluene/methanol 4/1 v/v (5 mL). The solution was stirred at room temperature and (TMS)CHN_2 (0.030 mL of 2M in hexane, 0.060 mmol) was added dropwise. The mixture was stirred for 1 h, after which the solution was diluted with 5 mL chloroform and 10 mL of 10 v% acetic acid aqueous solution. The aqueous layer was extracted with 10 mL chloroform and the organic fractions were combined. The organic phase was washed with saturated $NaHCO_3$, dried over Na_2SO_4, and concentrated in vacuo. The product was dried in the vacuum oven to yield a white solid material (0.024 g, 79 %). ^{1}H NMR (400 MHz, Chloroform-*d*) δ [ppm] = 8.29 – 8.21 (m, 3H), 7.53 (d, *J* = 7.6 Hz, 1H), 6.57 (t, *J* = 5.7 Hz, 2H), 4.78 – 4.73 (m, 1H), 4.16 (t, *J* = 6.8 Hz, 2H), 3.69 (s, 3H), 3.44 (q, *J* = 6.7 Hz, 4H), 2.58 – 2.45 (m, 2H), 2.40 – 2.28 (m, 1H), 2.23 – 2.10 (m, 1H), 1.72 – 1.55 (m, 6H), 1.43 – 1.18 (m, 38H), 0.88 (t, *J* = 6.9 Hz, 9H). ^{13}C NMR (100 MHz, Chloroform-*d*) δ [ppm] = 173.55, 171.91, 165.94, 165.75, 135.55, 134.27, 128.56, 127.96, 66.09, 52.70, 52.02, 40.42, 31.92, 31.82, 30.33, 29.66, 29.64, 29.59, 29.56, 29.51, 29.36, 29.31, 29.22, 28.53, 27.04, 26.89, 25.84, 22.69, 22.65, 14.12, 14.09. MALDI-TOF mass spectrometry: *m/z* ratio calculated for $[C_{43}H_{73}N_3O_7]$+([M·H]+): 744.54 ([M·H]+), observed: 744.58 ([M·H]+), 766.52 ([M·Na]+), 782.52 ([M·K]+).

Synthesis of (R)-Glu(OMe)-BTA

In the attempt to retrieve (*R*)-**4** (0.160 g, 0.20 mmol) from a failed deprotection attempt, the reaction mixture was dissolved in MeOH following removement of the solvent and TFA by rotary evaporation. Surprisingly, the methylated product (*R*)-**Glu(OMe)-BTA** had partially been formed in low yield, which was separated from the starting material by flash chromatography (0.018 g, 12 %). ^{1}H NMR (400 MHz, Chloroform-*d*) δ [ppm] = 8.29 (d, *J* = 5.6 Hz, 3H), 7.44 (d, *J* = 7.5 Hz, 1H), 6.50 (t, *J* = 5.8 Hz, 2H), 4.79 – 4.75 (m, 1H), 4.17 (t, *J* = 6.8 Hz, 2H), 3.70 (s, 3H), 3.45 (q, *J* = 6.7 Hz, 4H), 2.61 – 2.45 (m, 2H), 2.38 – 2.29 (m, 1H), 2.21 – 2.11 (m, 1H), 1.77 – 1.53 (m, 6H), 1.45 – 1.15 (m, 38H), 0.88 (t, *J* = 6.6 Hz, 9H). ^{13}C NMR (100 MHz, Chloroform-*d*) δ [ppm] = 173.55, 171.91, 165.94, 165.75, 135.55, 134.27, 128.56, 127.96, 66.09, 52.70,

52.02, 40.42, 31.92, 31.82, 30.33, 29.66, 29.64, 29.59, 29.56, 29.51, 29.36, 29.31, 29.22, 28.53, 27.04, 26.89, 25.84, 22.69, 22.65, 14.12, 14.09. MALDI-TOF mass spectrometry: *m/z* ratio calculated for $[C_{43}H_{73}N_3O_7]^+$([M·H]+): 744.54 ([M·H]+), observed: 744.58 ([M·H]+), 766.52 ([M·Na]+), 782.52 ([M·K]+).

Computational analysis of one-, two- and three-component supramolecular polymerizations

1. Details mass-balance model for supramolecular polymerizations

Following the derivation of cooperative polymerization with a nucleus size of 2 outlined by Zhao and Moore[2] the equivalent concentration of monomers in supramolecular polymers of length *i* ($[P_i]$) can be calculated from the free monomer concentration [M]:

$$[\mathrm{P}_i] = i \cdot \sigma \cdot K^{i-1} \cdot [\mathrm{M}]^i \text{ for } i \geq 2 \tag{S1}$$

Where σ is the cooperativity parameter, which is a ratio of the binding constant of nucleation (K_n) and elongation (K):

$$\sigma = \frac{K_\mathrm{n}}{K} \tag{S2}$$

In this model, the van 't Hoff equation is used to introduce the temperature-dependency of the binding constants:

$$K = \exp\left(\frac{-\Delta G}{R \cdot T}\right) = \exp\left(\frac{-\Delta H}{R \cdot T} + \frac{\Delta S}{R}\right) \tag{S3}$$

With ΔG the Gibbs free energy of elongation, R the gas constant, T the absolute temperature, ΔH the enthalpy of elongation and ΔS the entropy of elongation.

The enthalpy of nucleation was calculated by including the nucleation penalty (*NP*):

$$\Delta H_\mathrm{n} = \Delta H + NP \tag{S4}$$

The binding constant of nucleation at each temperature was calculated with the van 't Hoff equation.

The mass-balance equation for the supramolecular polymerization can be obtained by summing equation S1 for all aggregate sizes *i* and then expressing the total concentration of the monomer in the system as a function of the free monomer concentration:

$$[\mathrm{M}]_\mathrm{tot} = (1-\sigma) \cdot [\mathrm{M}] + \frac{\sigma \cdot [\mathrm{M}]}{(1 - K \cdot [\mathrm{M}])^2} \tag{S5}$$

A binary search algorithm is used in the model to solve the mass-balance equation.

From the speciation of the monomer, the simulated CD signal can be calculated by multiplying the amount of monomer in species i with the molar ellipticity from the corresponding species, θ_i. The molar ellipticity of monomeric species is set at 0 and the molar ellipticity of BTAs in aggregated species is estimated at $1.5 \cdot 10^6$ mdeg·$M^{-1}cm^{-1}$.

$$\mathrm{CD}_{\mathrm{norm,calc}} = \frac{\theta_p \cdot [\mathrm{M}]_p + \theta_m \cdot [\mathrm{M}]}{[\mathrm{M}]_{tot}} \tag{S6}$$

2. Details mass-balance model for two-component copolymerization (sergeant-and-soldiers and majority-rules)

To study the supramolecular copolymerization of two monomers **A** and **B**, the derivation of the mass-balance model was followed, as reported by Markvoort and co-workers.[3] The mass-balance equations for **A** and **B** monomers become:

$$[\mathbf{A}]_{tot} = [\mathbf{A}] + P_{\mathbf{A}}([\mathbf{A}],[\mathbf{B}]) \tag{S7}$$

$$[\mathbf{B}]_{tot} = [\mathbf{B}] + P_{\mathbf{B}}([\mathbf{A}],[\mathbf{B}]) \tag{S8}$$

Where $P_X([\mathbf{A}],[\mathbf{B}])$ for X as either **A** or **B** is the sum of the third and fourth component of the vector $\mathbf{U}^x$:

$$\mathbf{U}^{\mathrm{X}} = (I - M_{\mathrm{X}})^{-1} \cdot M_{\mathrm{X}} \cdot \mathbf{u}_1^{\mathrm{X}} \tag{S9}$$

With 4 x 4 identity matrix I, and $\mathbf{u}_1$:

$$\mathbf{u}_1^{\mathbf{A}} = \begin{pmatrix} \sigma_{\mathbf{A}} \cdot [\mathbf{A}] \\ \sigma_{\mathbf{B}} \cdot [\mathbf{B}] \\ \sigma_{\mathbf{A}} \cdot [\mathbf{A}] \\ 0 \end{pmatrix} \quad \text{or} \quad \mathbf{u}_1^{\mathbf{B}} = \begin{pmatrix} \sigma_{\mathbf{A}} \cdot [\mathbf{A}] \\ \sigma_{\mathbf{B}} \cdot [\mathbf{B}] \\ 0 \\ \sigma_{\mathbf{B}} \cdot [\mathbf{B}] \end{pmatrix} \tag{S10}$$

And M_X:

$$M_{\mathrm{X}} = \begin{pmatrix} K_{\mathbf{A\text{-}A}} \cdot [\mathbf{A}] & K_{\mathbf{B\text{-}A}} \cdot [\mathbf{A}] & 0 & 0 \\ K_{\mathbf{A\text{-}B}} \cdot [\mathbf{B}] & K_{\mathbf{B\text{-}B}} \cdot [\mathbf{B}] & 0 & 0 \\ K_{\mathbf{A\text{-}A}} \cdot [\mathbf{A}] \cdot \delta_{\mathbf{A\text{-}A}} & K_{\mathbf{B\text{-}A}} \cdot [\mathbf{A}] \cdot \delta_{\mathbf{B\text{-}A}} & K_{\mathbf{A\text{-}A}} \cdot [\mathbf{A}] & K_{\mathbf{B\text{-}A}} \cdot [\mathbf{A}] \\ K_{\mathbf{A\text{-}B}} \cdot [\mathbf{B}] \cdot \delta_{\mathbf{A\text{-}B}} & K_{\mathbf{B\text{-}B}} \cdot [\mathbf{B}] \cdot \delta_{\mathbf{B\text{-}B}} & K_{\mathbf{A\text{-}B}} \cdot [\mathbf{B}] & K_{\mathbf{B\text{-}B}} \cdot [\mathbf{B}] \end{pmatrix} \tag{S11}$$

Where $\delta_{\mathbf{A\text{-}A}} = \delta_{\mathbf{A\text{-}B}} = 1$ and $\delta_{\mathbf{B\text{-}A}} = \delta_{\mathbf{B\text{-}B}} = 0$ for X = **A** and $\delta_{\mathbf{A\text{-}A}} = \delta_{\mathbf{A\text{-}B}} = 0$ and $\delta_{\mathbf{B\text{-}A}} = \delta_{\mathbf{B\text{-}B}} = 1$ for X = **B.** The mass-balance equations are solved in the model with a nested binary search algorithm.

The simulated CD signal was calculating by using the sum of Eq. S6 for both **A** and **B** (Eq. S12). In other words, we assume that the molar ellipticity of each monomer is the same in the copolymer as in the homopolymers.

$$\mathrm{CD}_{\text{norm,calc}}(T) = \frac{\theta_{\mathbf{A},p} \cdot [\mathbf{A}]_p + \theta_{\mathbf{A},m} \cdot [\mathbf{A}] + \theta_{\mathbf{B},p} \cdot [\mathbf{B}]_p + \theta_{\mathbf{B},m} \cdot [\mathbf{B}]}{[\mathbf{A}]_{tot} + [\mathbf{B}]_{tot}} \tag{S12}$$

3. Details mass-balance model for the three-component copolymerization (mixed majority-rules)

To study the supramolecular copolymerization of three components in mixed majority-rules systems, the two-component copolymerization model was extended with an additional component **C**. The mass-balance equations for monomer **A**, **B**, and **C** become:

$$[\mathbf{A}]_{tot} = [\mathbf{A}] + P_{\mathbf{A}}([\mathbf{A}],[\mathbf{B}],[\mathbf{C}]) \tag{S13}$$

$$[\mathbf{B}]_{tot} = [\mathbf{B}] + P_{\mathbf{B}}([\mathbf{A}],[\mathbf{B}],[\mathbf{C}]) \tag{S14}$$

$$[\mathbf{C}]_{tot} = [\mathbf{C}] + P_{\mathbf{C}}([\mathbf{A}],[\mathbf{B}],[\mathbf{C}]) \tag{S15}$$

Where P_X([**A**],[**B**],[**C**]) for X as either **A** or **B** or **C** is the sum of the third and fourth component of the vector $\mathbf{U}^x$ (Eq. S9) with 4 x 4 matrix I and $\mathbf{u}_1$:

$$\mathbf{u}_1^{\mathbf{A}} = \begin{pmatrix} \sigma_{\mathbf{A}} \cdot [\mathbf{A}] \\ \sigma_{\mathbf{B}} \cdot [\mathbf{B}] \\ \sigma_{\mathbf{C}} \cdot [\mathbf{C}] \\ \sigma_{\mathbf{A}} \cdot [\mathbf{A}] \\ 0 \\ 0 \end{pmatrix} \quad \text{or} \quad \mathbf{u}_1^{\mathbf{B}} = \begin{pmatrix} \sigma_{\mathbf{A}} \cdot [\mathbf{A}] \\ \sigma_{\mathbf{B}} \cdot [\mathbf{B}] \\ \sigma_{\mathbf{C}} \cdot [\mathbf{C}] \\ 0 \\ \sigma_{\mathbf{B}} \cdot [\mathbf{B}] \\ 0 \end{pmatrix} \quad \text{or} \quad \mathbf{u}_1^{\mathbf{C}} = \begin{pmatrix} \sigma_{\mathbf{A}} \cdot [\mathbf{A}] \\ \sigma_{\mathbf{B}} \cdot [\mathbf{B}] \\ \sigma_{\mathbf{C}} \cdot [\mathbf{C}] \\ 0 \\ 0 \\ \sigma_{\mathbf{C}} \cdot [\mathbf{C}] \end{pmatrix} \tag{S16}$$

And M_X:

$$M_{\mathrm{X}} = \begin{pmatrix} K_{\mathbf{A\text{-}A}} \cdot [\mathbf{A}] & K_{\mathbf{B\text{-}A}} \cdot [\mathbf{A}] & K_{\mathbf{C\text{-}A}} \cdot [\mathbf{A}] & 0 & 0 & 0 \\ K_{\mathbf{A\text{-}B}} \cdot [\mathbf{B}] & K_{\mathbf{B\text{-}B}} \cdot [\mathbf{B}] & K_{\mathbf{C\text{-}B}} \cdot [\mathbf{B}] & 0 & 0 & 0 \\ K_{\mathbf{A\text{-}C}} \cdot [\mathbf{B}] & K_{\mathbf{B\text{-}C}} \cdot [\mathbf{B}] & K_{\mathbf{C\text{-}C}} \cdot [\mathbf{C}] & 0 & 0 & 0 \\ K_{\mathbf{A\text{-}A}} \cdot [\mathbf{A}] \cdot \delta_{\mathbf{A\text{-}A}} & K_{\mathbf{B\text{-}A}} \cdot [\mathbf{A}] \cdot \delta_{\mathbf{B\text{-}A}} & K_{\mathbf{C\text{-}A}} \cdot [\mathbf{A}] \cdot \delta_{\mathbf{C\text{-}A}} & K_{\mathbf{A\text{-}A}} \cdot [\mathbf{A}] & K_{\mathbf{B\text{-}A}} \cdot [\mathbf{A}] & K_{\mathbf{C\text{-}A}} \cdot [\mathbf{A}] \\ K_{\mathbf{A\text{-}B}} \cdot [\mathbf{B}] \cdot \delta_{\mathbf{A\text{-}B}} & K_{\mathbf{B\text{-}B}} \cdot [\mathbf{B}] \cdot \delta_{\mathbf{B\text{-}B}} & K_{\mathbf{C\text{-}B}} \cdot [\mathbf{B}] \cdot \delta_{\mathbf{C\text{-}B}} & K_{\mathbf{A\text{-}B}} \cdot [\mathbf{B}] & K_{\mathbf{B\text{-}B}} \cdot [\mathbf{B}] & K_{\mathbf{C\text{-}B}} \cdot [\mathbf{B}] \\ K_{\mathbf{A\text{-}C}} \cdot [\mathbf{C}] \cdot \delta_{\mathbf{A\text{-}C}} & K_{\mathbf{B\text{-}C}} \cdot [\mathbf{C}] \cdot \delta_{\mathbf{B\text{-}C}} & K_{\mathbf{C\text{-}C}} \cdot [\mathbf{C}] \cdot \delta_{\mathbf{C\text{-}C}} & K_{\mathbf{A\text{-}C}} \cdot [\mathbf{C}] & K_{\mathbf{B\text{-}C}} \cdot [\mathbf{C}] & K_{\mathbf{C\text{-}C}} \cdot [\mathbf{C}] \end{pmatrix}$$

(S17)

Where $\delta_{\mathbf{A}\text{-}\mathbf{A}} = \delta_{\mathbf{A}\text{-}\mathbf{B}} = \delta_{\mathbf{A}\text{-}\mathbf{C}} = 1$ and $\delta_{\mathbf{B}\text{-}\mathbf{A}} = \delta_{\mathbf{B}\text{-}\mathbf{B}} = \delta_{\mathbf{B}\text{-}\mathbf{C}} = \delta_{\mathbf{C}\text{-}\mathbf{A}} = \delta_{\mathbf{C}\text{-}\mathbf{B}} = \delta_{\mathbf{C}\text{-}\mathbf{C}} = 0$ for X = **A** and $\delta_{\mathbf{B}\text{-}\mathbf{A}} = \delta_{\mathbf{B}\text{-}\mathbf{B}} = \delta_{\mathbf{B}\text{-}\mathbf{C}} = 1$ and $\delta_{\mathbf{A}\text{-}\mathbf{A}} = \delta_{\mathbf{A}\text{-}\mathbf{B}} = \delta_{\mathbf{A}\text{-}\mathbf{C}} = \delta_{\mathbf{C}\text{-}\mathbf{A}} = \delta_{\mathbf{C}\text{-}\mathbf{B}} = \delta_{\mathbf{C}\text{-}\mathbf{C}} = 0$ for X = **B** and $\delta_{\mathbf{C}\text{-}\mathbf{A}} = \delta_{\mathbf{C}\text{-}\mathbf{B}} = \delta_{\mathbf{C}\text{-}\mathbf{C}} = 1$ and $\delta_{\mathbf{A}\text{-}\mathbf{A}} = \delta_{\mathbf{A}\text{-}\mathbf{B}} = \delta_{\mathbf{A}\text{-}\mathbf{C}} = \delta_{\mathbf{B}\text{-}\mathbf{A}} = \delta_{\mathbf{B}\text{-}\mathbf{B}} = \delta_{\mathbf{B}\text{-}\mathbf{C}} = 0$ for X = **C**.

The mass-balance equations are solved in the model with a nested binary search algorithm.

The simulated CD signal was calculated by using the sum of Eq. S6 for each comonomer (Eq. S18). In other words, we assume that the molar ellipticity of each monomer is the same in the copolymer as in the homopolymers.

$$\mathrm{CD}_{\mathrm{norm,calc}}(T) = \frac{\theta_{\mathbf{A},p}\cdot[\mathbf{A}]_p + \theta_{\mathbf{A},m}\cdot[\mathbf{A}] + \theta_{\mathbf{B},p}\cdot[\mathbf{B}]_p + \theta_{\mathbf{B},m}\cdot[\mathbf{B}] + \theta_{\mathbf{C},p}\cdot[\mathbf{C}]_p + \theta_{\mathbf{C},m}\cdot[\mathbf{C}]}{[\mathbf{A}]_{tot} + [\mathbf{B}]_{tot} + [\mathbf{C}]_{tot}} \quad \text{(S18)}$$

Optimization of Glu-BTA molecular design

The **Glu-BTA** molecular design was optimized to match conditions with respect to assembly and reactivity. We started by screening commercially available methylating and acylating reagents on organic model compounds bearing polar moieties (-OH, -COOOH, $-NH_2$) in apolar solvent and dilute reaction condition. These model compounds were lauric acid, dodecanol and dodecylamine with N-methylacetamide as a control compound to check reactivity towards the amide moiety. The conversions were monitored with ^{1}H NMR. Reagents tested were methyl iodide in combination with a base (tested were NaH, DBU, KHMDS, 'proton sponge' and triethylamine), methyl trifluoromethanesulfonate ('methyl triflate'), (trimethylsilyl)diazomethane ((TMS)CHN_2), acetyl chloride and acetic anhydride. In this screening stage, we found most promising results (*i.e.* good conversion and specificity, no precipitation) for the methylation of carboxylic acid with (TMS)CHN_2 (see Figure S1), of which further optimization for *in situ* reaction application is discussed later in this supporting information.

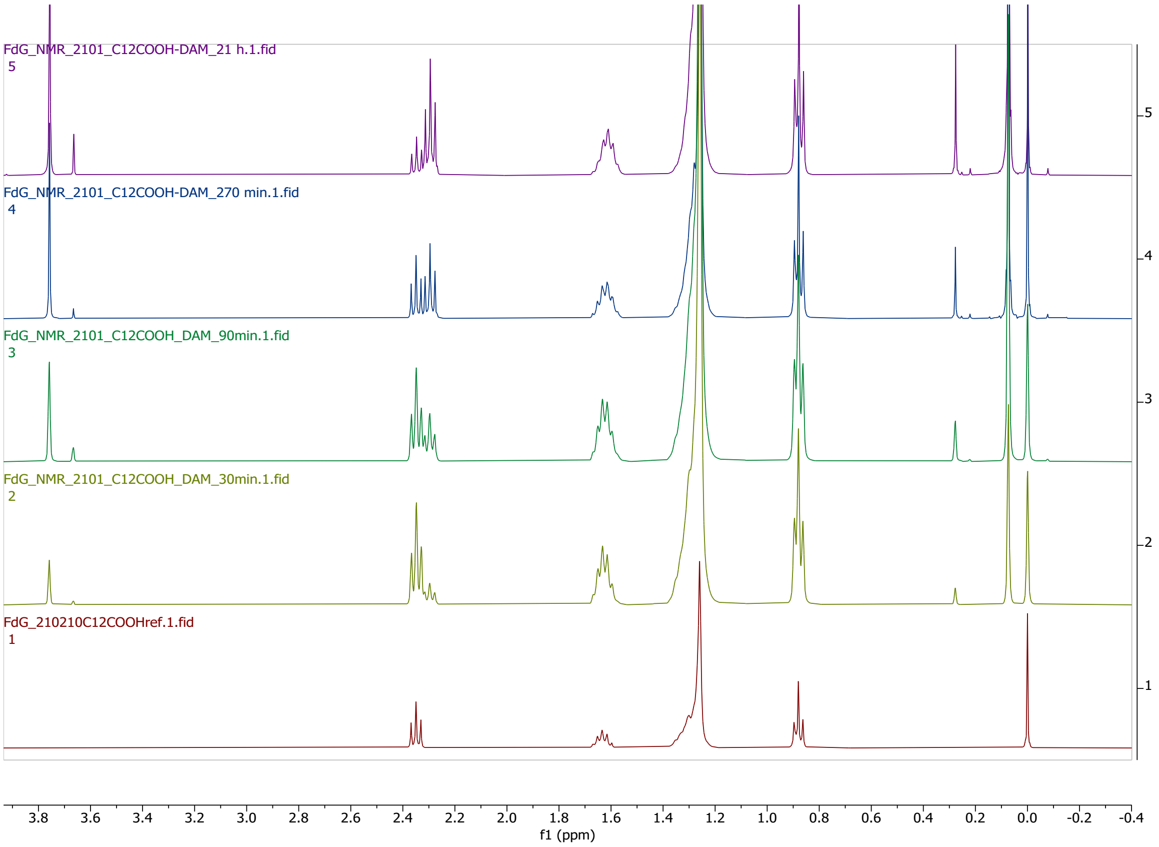

Figure S1: Collection of ^{1}H NMR measurements of a 1 mM solution of lauric acid with 20 equivalents of (TMS)CHN_2. The two signals at 3.66 and 3.75 ppm correspond to the methyl-ester reaction products. From bottom to top: lauric acid reference spectrum, reaction mixture lauric acid + (TMS)CHN_2 after 30 minutes, 90 minutes, 270 minutes, 21 hours.

Following our initial screening, we synthesized the glutamic acid side chain via amidation of dodecylamine to Z-Glu(OtBu)-OH to obtain Z-Glu(OtBu)-$NHC_{12}H_{25}$. We chose dodecylamine over dodecanol for the higher nucleophilicity of the amine, aiming at high yields. Next, we synthesized the trisubstituted BTA from trimesic acid with H_2N-Glu(OtBu)-$NHC_{12}H_{25}$ and studied the BTA product (*tert*-butyl protected) with CD spectroscopy at 50 µM in MCH (Figure S2a). The CD spectrum showed a typical shape that reveals the formation of dimeric aggregates, which were stable up to at least 90 °C.[4] Deprotection of the *tert*-butyl moiety with trifluoroacetic acid (TFA) to yield the carboxylic acid resulted in a molecule that was insoluble in apolar solvents. We acted accordingly by changing the design to an asymmetric BTA and coupling H_2N-Glu(OtBu)-$NHC_{12}H_{25}$ to compound **3**. The obtained precursor $(C_8H_{17})_2$-BTA-Glu(OtBu)-$NHC_{12}H_{25}$ showed a single positive CD band at room temperature and a deviating CD spectrum at 90 °C (Figure S2b). Deprotection of the *tert*-butyl moiety resulted in a molecule that solubilized in MCH at high temperature but precipitated upon cooling. We speculated that side interactions originating from the amide connecting the dodecyl chain with the glutamic acid might be problematic for 1D polymer assembly, therefore we replaced this amide with an ester (See synthesis of **Glu-BTA**). We kept the asymmetric design to diminish the morphological transition from dimers to fibers which has been shown for symmetric ester-BTAs.[4,5] The

obtained **Glu-BTA** (Scheme 1, main text) showed a desired solubility in MCH both at elevated and room temperature.

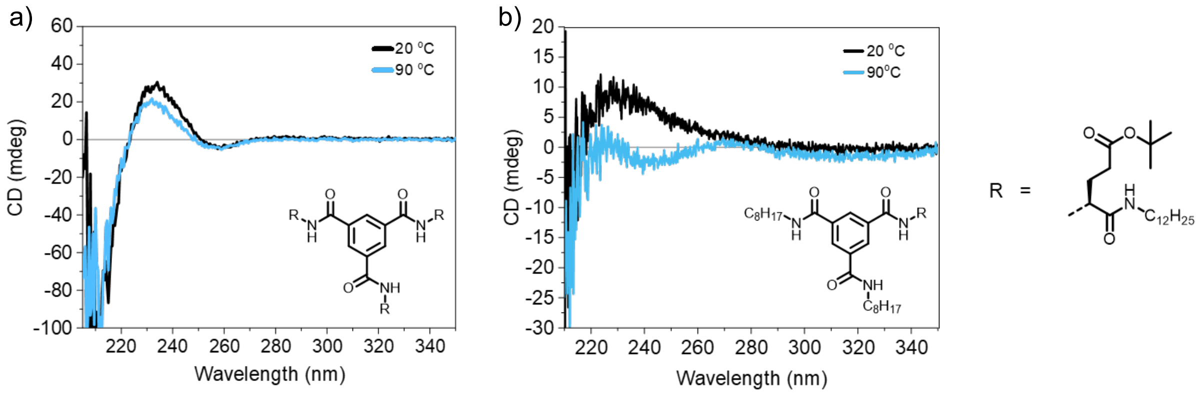

Figure S2: CD spectra of the symmetric (a) and asymmetric (b) amide-BTAs before deprotection of the *tert*-butyl moiety at 90 °C (blue) and 20 °C (black). Measurements were done at 50 µM in MCH.

Additional figures on the characterization of supramolecular homopolymers

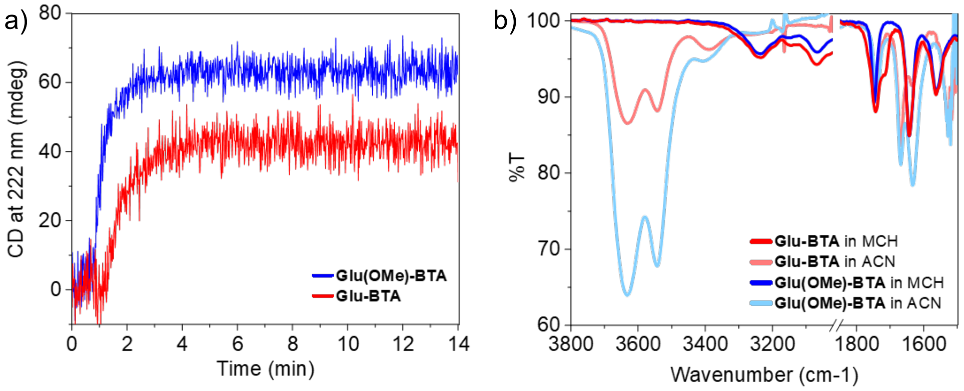

Figure S3: (a) Kinetic evolution experiments of freshly prepared 50 µM solutions of **Glu-BTA** (red) and **Glu(OMe)-BTA** (blue) in MCH. For these measurements, the samples in quartz cuvettes were equilibrated at 90 °C in a water bath close to the CD spectrophotometer, of which the cell holder was equilibrated at 20 °C. After equilibration of 15 minutes, the time course measurement was started, and the samples were directly transferred from the hot water bath into the cell holder. The kinetic measurement was followed over 15 minutes with data intervals of 1 second. (b) FTIR spectra at 1 mM of **Glu-BTA** and **Glu(OMe)-BTA**. In MCH, **Glu-BTA** (red) and **Glu(OMe)-BTA** (dark blue) show similar spectra with absorption bands at 3236 cm^{-1} (bonded N-H stretch), 1643 cm^{-1} (bonded C=O amide I vibrational mode) and 1562 cm^{-1} (bonded C=O amide II vibrational mode) that are typical for threefold hydrogen bonding between amides at the BTA core. In acetonitrile, for both **Glu-BTA** (pink) and **Glu(OMe)-BTA** (light blue) these typical absorption bands are absent.

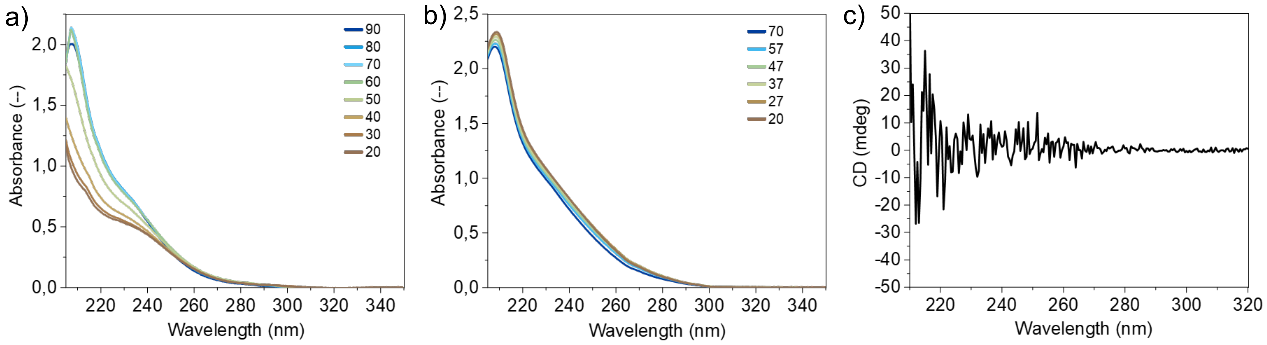

Figure S4: 50 µM samples of **Glu(OMe)-BTA**. UV-vis absorption spectra at varying temperatures in MCH (a) and acetonitrile (b). The hypsochromic shifts observed in MCH (a) are a sign of polymeric aggregation, which are not observed in acetonitrile (b). (c) The CD spectrum in acetonitrile at 20 °C. The absence of a CD signal indicates that there is no formation of homochiral helical supramolecular polymers.

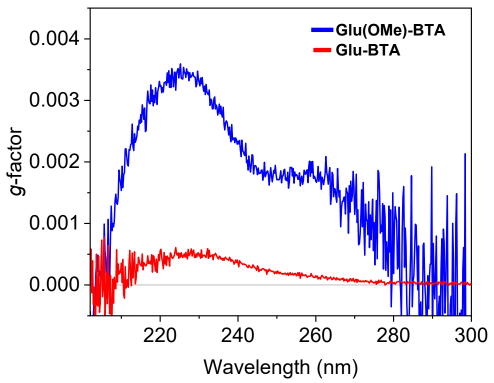

Figure S5: Calculated g-factors for **Glu-BTA** (red) and **Glu(OMe)-BTA** (blue) at 50 µM, 20 °C in MCH according to g-factor = (CD/33000)/Absorption.

Thermodynamic analysis of homopolymers

1. Van 't Hoff analysis.

Both **Glu-BTA** and **Glu(OMe)-BTA** homopolymer systems were subjected to thermodynamic analysis for cooperative supramolecular polymerization. Such a polymerization can be described in terms of changes in enthalpy (ΔH) and entropy (ΔS), which can be obtained by analyzing obtained data with the Van 't Hoff equation (Eq. S3). For a cooperative polymerization, the concentration of free monomer ($[\mathbf{M}]_{free}$) is equal to the total concentration of monomer ($[\mathbf{M}]_{tot}$) at the temperature of elongation (T_e, Equation S19). In addition, the product of the equilibrium constant and the concentration of free monomer is equal to unity (Equation S20):

$$[\mathbf{M}]_{free} = [\mathbf{M}]_{tot} \text{ at } T = T_e \quad \text{(S19)}$$

$$K_e \times K' \times [\mathbf{M}]_{free} = 1 \text{ at } T = T_e \quad \text{(S20)}$$

With K' is a unit equilibrium (1 L/mol). Substitution of Equations S19 and S20 into S3 yields Equation S21:

$$\ln\left(c'/[\mathbf{M}]_{tot}\right) = \left(-\Delta H/R\right) \times \left(1/T_e\right) + \Delta S/R \qquad \text{(S21)}$$

Where c' is a unit concentration (1 mol/L). Note that the unit equilibrium constant and unit concentration are used to correct for the dimensions of the parameters in Equations S20 and S21, that is, the equilibrium constant has no dimension whereas the total concentration of monomer has dimension mol/L.

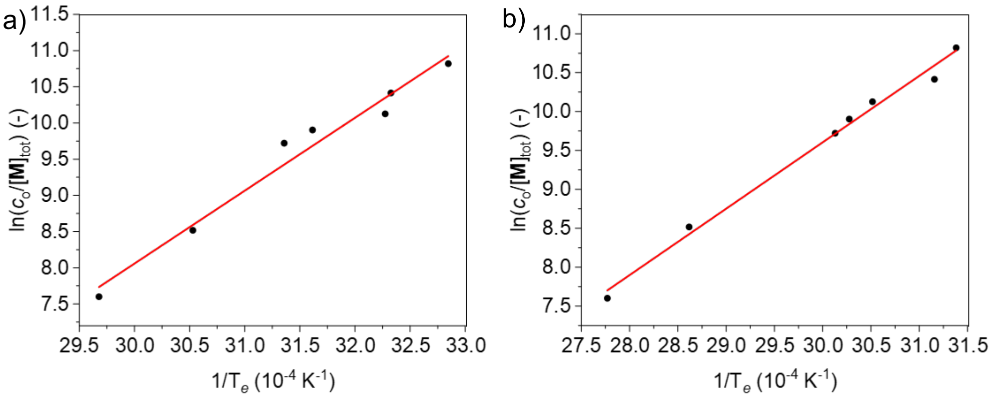

Figure S6: Van 't Hoff analyses of a) **Glu-BTA** and b) **Glu(OMe)-BTA** homopolymers obtained from CD experiments on solutions at concentrations ranging from 20 to 500 µM at 20 °C in MCH.

Table S1: Thermodynamic parameters describing the homopolymerizations of **Glu-BTA** and **Glu(OMe)-BTA**. The parameters were obtained from the Van 't Hoff analysis. Changes in Gibbs free energy of elongation (ΔG_e) are reported for 293 K.

Compound	ΔH_e (kJ mol^{-1})	ΔS_e (kJ mol^{-1} K^{-1})	ΔG_e (kJ mol^{-1})	K_e (10^4)
**Glu-BTA**	-83. 7	-0.184	-29.8	20.2
**Glu(OMe)-BTA**	-70.9	-0.133	-32.0	50.5

2. Thermodynamic parameters determined by fitting one-component equilibrium model to experimental data.

Fitting the one-component equilibrium mass-balance model to the CD data acquired for **Glu-BTA** and **Glu(OMe)-BTA** in the range of 20 to 60 µM allows to determine the Gibbs free energy of elongation and the nucleation penalty. However, the temperature-dependent data of **Glu(OMe)-BTA** showed a non-zero CD signal and non-flat UV-vis absorption signal at temperatures above the T_e, which could indicate formation of small aggregates, such as dimers, prior to polymerization. The mass-balance model does not take this into account. Therefore, the extracted thermodynamic parameters might be inaccurate, hence we do not solely rely on the thermodynamic parameters using this method but combine the results with the Van 't Hoff analysis.

The temperature-dependent CD data was corrected for concentration to weigh all samples equally in the fitting procedure. The fitting parameters were the entropy and enthalpy of elongation and the

nucleation penalty. The entropy was sampled between -0.050 and -0.150 kJ mol^{-1} K^{-1}, the Gibbs free energy between -10 and -40 kJ mol^{-1} to determine the enthalpy and the nucleation penalty between -5 and -40 kJ mol^{-1}. The lhsdesign function in Matlab R2019a was used to create 500 initial parameter sets using Latin hypercube sampling. Each of these initial parameter sets was optimized to minimize the sum of squares of the cost vector with the Levenberg-Marquardt algorithm. The Matlab function lsqnonlin was used to perform the minimization of the cost vector, selecting for the lowest norm of the residual sum of squares. The cost vector contains the differences between each normalized data point and the corresponding calculated normalized CD signal at temperature T:

$$\text{Cost} = \text{CD}_{\text{norm,calc}}(T) - \text{CD}_{\text{norm}}(T) \tag{S22}$$

The simulated CD signals in Eq. 22 are calculated according to Eq. S6 and are shown in Figure S7 below.

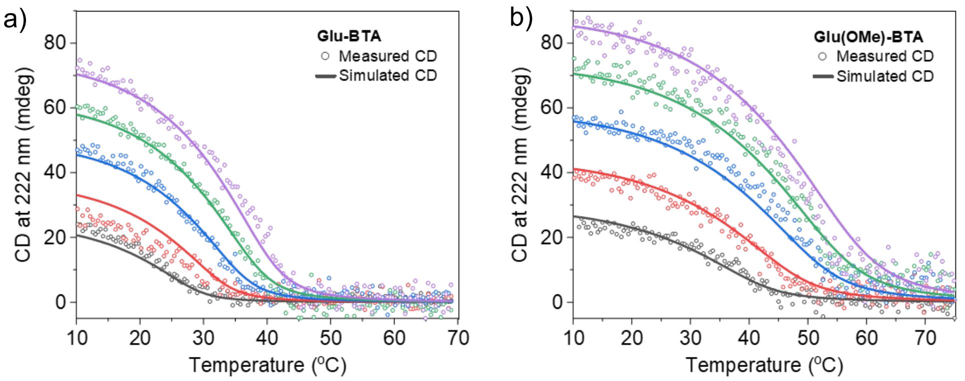

Figure S7: Experimental (circles) and simulated (lines) temperature-dependent CD measurements of the homopolymerizations of **Glu-BTA** (a) and **Glu(OMe)-BTA** (b) in MCH at various concentrations: 20 µM (grey), 30 µM (red), 40 µM (blue), 50 µM (green) and 60 µM (violet). Cooling rate: 1 °C/min. Optical path length: 1 cm.

Table S2: Thermodynamic parameters for homopolymerizations of **Glu-BTA** and **Glu(OMe)-BTA** extracted from fitting the experimental temperature-dependent CD data to the one-component mass-balance model. Changes in Gibbs free energy of elongation (ΔG_e), nucleation (ΔG_n) and the cooperativity factor (σ) are reported for 293 K.

Compound	ΔH_e (kJ mol^{-1})	ΔH_n (kJ mol^{-1})	ΔS_e (kJ mol^{-1} K^{-1})	ΔG_e (kJ mol^{-1})	ΔG_n (kJ mol^{-1})	σ (-)
**Glu-BTA**	-71.2	-60.8	-0.147	-28.0	-17.6	1.4×10^{-2}
**Glu(OMe)-BTA**	-64.7	-55.9	-0.117	-30.5	-21.6	2.7×10^{-2}

Additional figures on the characterization of supramolecular copolymers

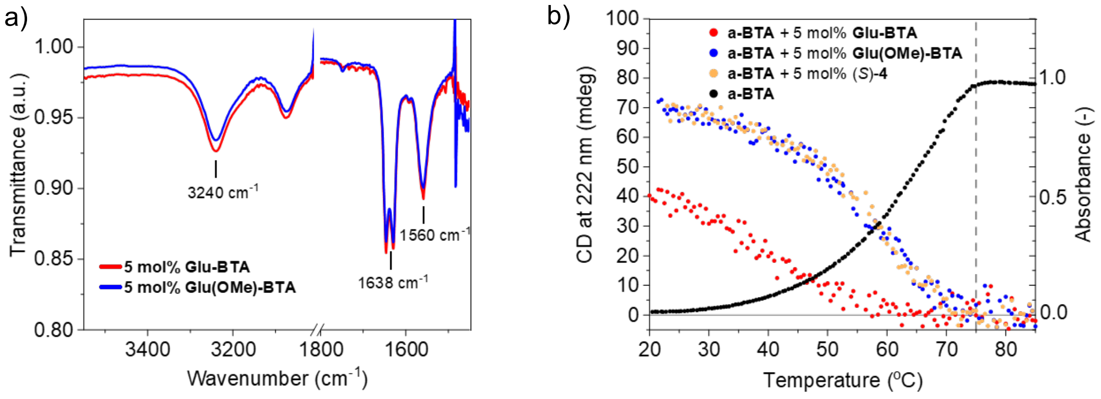

Figure S8: (a) FTIR measurements of 1 mM solutions of **a-BTA** containing 5 mol% of **Glu-BTA** (red) or **Glu(OMe)-BTA** (blue). The absorption bands at 3240 cm^{-1}, 1638 cm^{-1} and 1560 cm^{-1} confirm the polymerized state of the monomers in solution. No bands are observed near 3400 cm^{-1}, indicating no significant proportion of chain ends to ratio of monomers in stacks. (b) Temperature-dependent UV (**a-BTA**, black) and CD at 222 nm of mixtures containing 5 mol% of **Glu-BTA** (red), **Glu(OMe)-BTA** (blue) and **(*S*)-4** (yellow). The identical temperature-dependent data of sergeant **(*S*)-4** with respect to **Glu(OMe)-BTA** emphasizes the effect of the carboxylic acid moiety in the structure of sergeant **Glu-BTA** on the introduction of a helical bias to the soldiers' supramolecular polymers. All experiments were conducted at c_{tot} = 50 µM, 20 °C in MCH.

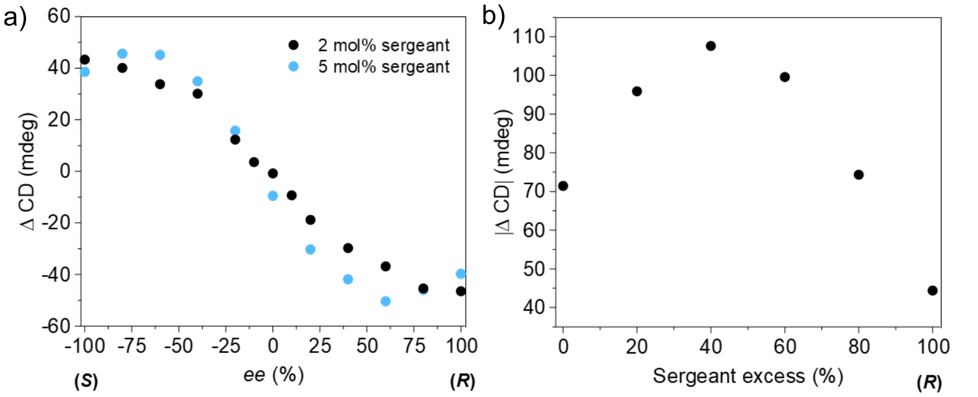

Figure S9: Plotted ΔCD values at 222 nm. (a) Between diluted majority-rules experiments of (*S*)-/(*R*)-**Glu-BTA** and (*S*)-/(*R*)-**Glu(OMe)-BTA** at $f_{sergeant}$ = 0.02 (black) and $f_{sergeant}$ = 0.05 (blue). The ΔCD at each *ee* is increased for the higher sergeant fraction compared to the lower sergeant fraction, except for *ee* = 100%. The increase in ΔCD is the result of a stronger nonlinear effect on the asymmetry by changes in the *ee* of **Glu(OMe)-BTA** while the effect of **Glu-BTA** remains linear over the increase in sergeant fraction. In addition, at 5 mol% of sergeants, a clear optimum of asymmetric amplification between enantiomeric mixtures of **Glu-BTA** and **Glu(OMe)-BTA** is observed at $ee_{sergeant}$ = 60 %. (b) Absolute ΔCD values between diluted majority-rules experiments of (*R*)-**Glu-BTA** with (*S*)-**Glu(OMe)-BTA** and (*S*)-/(*R*)-**Glu(OMe)-BTA** at $f_{sergeant}$ = 0.05. The plot shows that the highest attainable change in asymmetry can be reached by

conducting the *in situ* methylation in a system with 40% excess of (*R*)-**Glu-BTA** in the 5 mol% sergeant fraction. All experiments were conducted at c_{tot} = 50 μM, 20 °C in MCH.

Sergeant-and-soldiers simulations

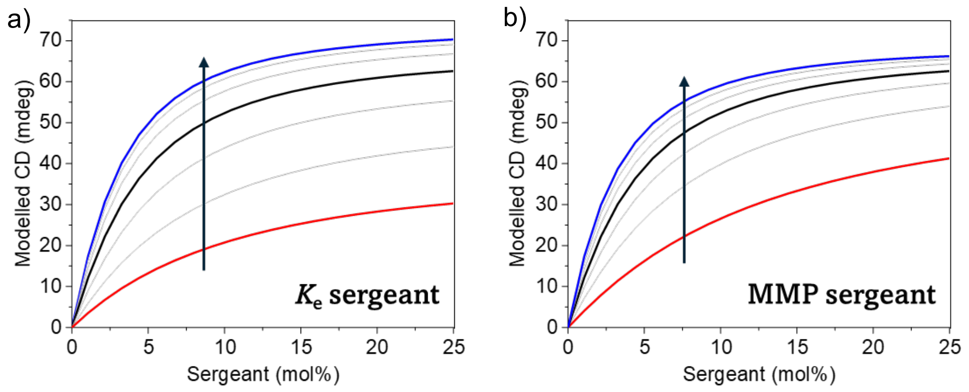

Figure S10: Simulated CD spectra of sergeant-and-soldiers experiments with variations in K_e (a, with MMP = 1.75 kJ mol^{-1}) and MMP (b, with $K_{e,sergeant}$ = 1.02 × 10^5). The color transition from red to blue represents the increase in K_e (a, range: 1.3 × 10^4 – 8.0 × 10^5) or MMP (b, range: 0.5 – 3.0 kJ mol^{-1}). The arrows are drawn to clarify the effect of increasing value on the net helicity. $f_{sergeant}$ = 0.05, $\sigma_{soldier}$ = 0.026, $\sigma_{sergeant}$ = 0.016, $K_{e,soldier}$ = 1.54×10^6.

Majority-rules experiments

The majority-rules experiments were conducted to verify whether **Glu-BTA** or **Glu(OMe)-BTA** showed stronger nonlinear translation of asymmetry over the range of *ee*. To assess this, the nonlinearity factor from which the signal at *ee* = 40 % deviates from a linear regime was determined. A higher nonlinearity factor implies a stronger nonlinearity on asymmetry over the *ee* range. The expected signal in a linear regime was determined following Eq. S23:

$$CD_{lin,40\%} = CD_{100\%}/100 \times 40 \quad (S23)$$

The nonlinearity factor was then determined by dividing the outcome of Eq. S23 by the measured CD value at *ee* = 40 %:

$$F_{nonlin} = CD_{40\%} \,/\, CD_{lin,40\%} \quad (S24)$$

F_{nonlin} was determined at 1.13 for **Glu-BTA** and at 2.12 for **Glu(OMe)-BTA**. Hence, stronger nonlinearity was observed in the asymmetry over the range of *ee* for **Glu(OMe)-BTA** compared to **Glu-BTA**.

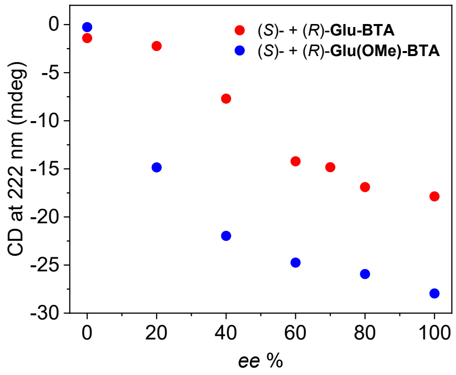

Figure S11: Majority rules experiments of **Glu-BTA** (red) and **Glu(OMe)-BTA** (blue) (*ee* range: 0 % to 100 % (*R*)-enantiomer) at 20 μM, 10 °C in MCH. The experiments were conducted at 20 μM and 10 °C to diminish interfering interactions of the carboxylic acid-moiety of **Glu-BTA** on the asymmetry.

Optimization of the *in situ* methylation procedure

Following the identification of $(TMS)CHN_2$ as an effective and selective methylating agent for the carboxylic acid moiety (*vide supra*), quantitative conversion of **Glu-BTA** into **Glu(OMe)-BTA** was achieved following literature procedure.[6] This procedure involved high amounts of methanol which will be harmful for the supramolecular polymers in solution. Fortunately, it was found that HBF_4 acts as a catalyst in the methyl transfer on the methylation of carboxylic acids, allowing us to lower the amount of methanol to a tolerable volume.[7] With lauric acid as a model compound, we conducted experiments in cyclohexane in which we varied the concentration of lauric acid, the amount of methanol, the amount of HBF_4 and the amount of $(TMS)CHN_2$ and monitored the conversion of the carboxylic acid into the methyl ester. Here, we found that concentration of the substrate, the amount of HBF_4 and the amount of $(TMS)CHN_2$ have the largest effect on the conversion.

Our initial attempts to *in situ* convert **Glu-BTA** into **Glu(OMe)-BTA** while monitoring the CD effect was for homopolymers of **Glu-BTA** at 50 μM (optical path length = 1 cm) and 200 μM (optical path length = 1 mm) in MCH. Addition of $(TMS)CHN_2$ to these solutions of **Glu-BTA** showed interference in the UV-vis absorption range where the CD effect for the BTAs is observed (220 – 250 nm), limiting the amount of $(TMS)CHN_2$ that we could add to the solution in order to monitor assembly. For a 50 μM solution of **Glu(OMe)-BTA** in MCH, 0.05 v% methanol was the maximum tolerated amount without complete loss of CD signal (Figure S12a). The loss of CD signal was only marginal at 0.03 v% methanol in MCH, which was therefore identified as an ideal solvent composition in the trade-off for reactivity and assembly. From the solvent composition of MeOH 0.03 v% in MCH, the tolerance of **Glu(OMe)-BTA** towards addition of HBF_4 was tested. Up to 2 equivalents of HBF_4 with respect to **Glu(OMe)-BTA** (50 μM) was tolerated without significant loss of CD signal (Figure S12b). As HBF_4 only has to be added in catalytic amounts, we did not intend to go this high in HBF_4 loading.

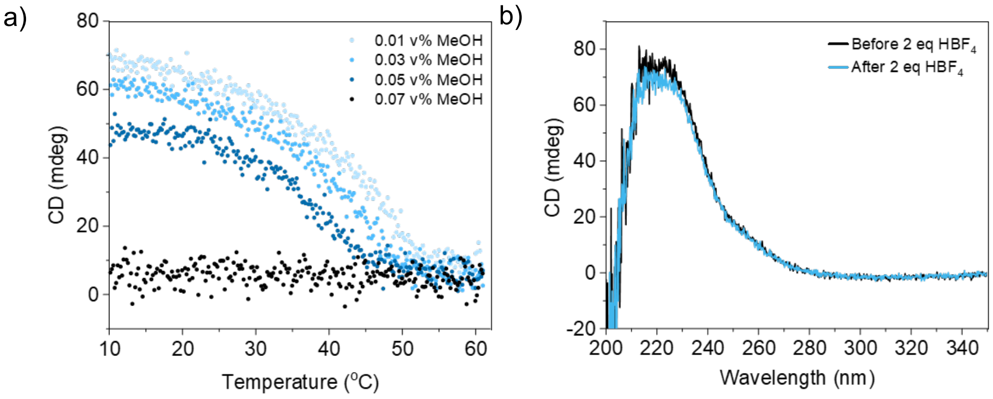

Figure S12: 50 µM solutions of **Glu(OMe)-BTA** in MCH at 20 °C. (a) Temperature-dependent CD data with increasing volume% of methanol from 0.01 to 0.07 v%. (b) Solution containing 0.03 v% methanol before (black) and after (blue) the addition of 2 equivalents of HBF_4.

At 50 µM of **Glu-BTA**, we conducted *in situ* methylation experiments while monitoring the CD at 222 nm with variations in HBF_4 loading (0.2 – 1 equivalents), methanol volume (0.03-0.06 v%) and $(TMS)CHN_2$ loading (2-30 equivalents). None of the experiments showed an obvious increase in CD signal over time. This can be ascribed due to the similar CD signal that is produced by both **Glu-BTA** and **Glu(OMe)-BTA** supramolecular polymers, together with the high noise in the signal that is caused by addition of $(TMS)CHN_2$. However, also at elevated temperatures where no assembly of **Glu-BTA** is observed, there was no increase in the CD observed after the addition of reagents. For one experiment of 50 µM **Glu-BTA** with 0.1 v% methanol (fully disrupting all supramolecular polymers), we added 0.2 equivalents of HBF_4 and 10 equivalents of $(TMS)CHN_2$ with respect to **Glu-BTA** and equilibrated the solution overnight. The solvent was removed by rotary evaporation and MCH was added to gain back the 50 µM solution, such that methanol was removed. The temperature-dependent CD data at 222 nm was measured and compared to the data of **Glu-BTA** and **Glu(OMe)-BTA**, revealing a T_e of the mixture that is situated in between the T_e's of **Glu-BTA** and **Glu(OMe)-BTA** indicating partial conversion.

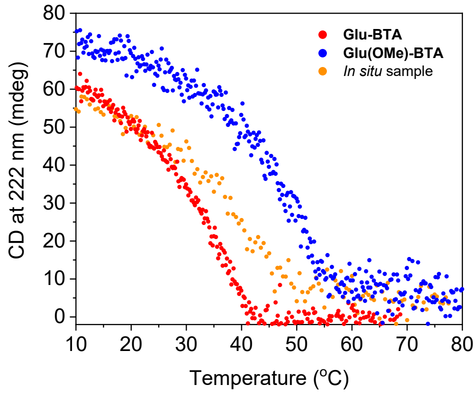

Figure S13: Temperature-dependent CD data of 50 µM solutions in MCH of **Glu-BTA** (red), **Glu(OMe)-BTA** (blue) and the *in situ* sample (orange) after refreshment of the solvent.

We then focused on the optimization of the reaction in a sergeant-and-soldier system. As a consequence, the substrate (*i.e.* the **Glu-BTA**) concentration was reduced from 50 μM to 1 – 2.5 μM. This allowed for higher loadings of (TMS)CHN_2 and HBF_4 with respect to the sergeant without harming supramolecular polymers formed by the soldiers. For all *in situ* methylation experiments in sergeant-and-soldier systems, we added a constant amount of HBF_4 (1 – 2 equivalents relative to the sergeant) and (TMS)CHN_2 (60 – 120 equivalents relative to the sergeant). The effect of reagents on the stability of the polymers could be monitored by stepwise addition of each reagent. Figure S14 shows an example of the CD time course upon addition of MeOH, after which the CD slightly drops due to destabilization of the polymers. Addition of the small quantity of HBF_4 has little influence, and upon addition of (TMS)CHN_2 the signal increases due to the conversion into **Glu(OMe)-BTA**. High conversions resulted compared to what was observed in homosystems of **Glu-BTA**.

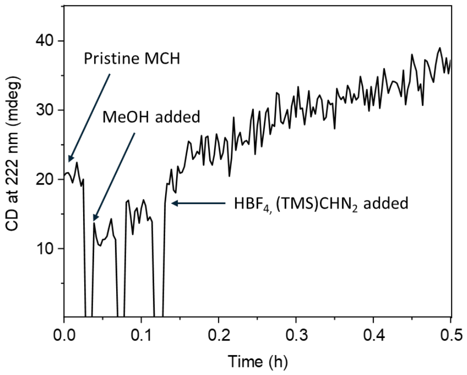

Figure S14: CD time course measurement of the *in situ* conversion of 2 mol% **Glu-BTA** in a 50 μM sample in MCH. Addition of MeOH to the pristine solution in MCH results in a drop in the CD value from 20 to 12 mdeg. The signal increases again after the addition of (TMS)CHN_2.

^{1}H NMRs

(S)-2

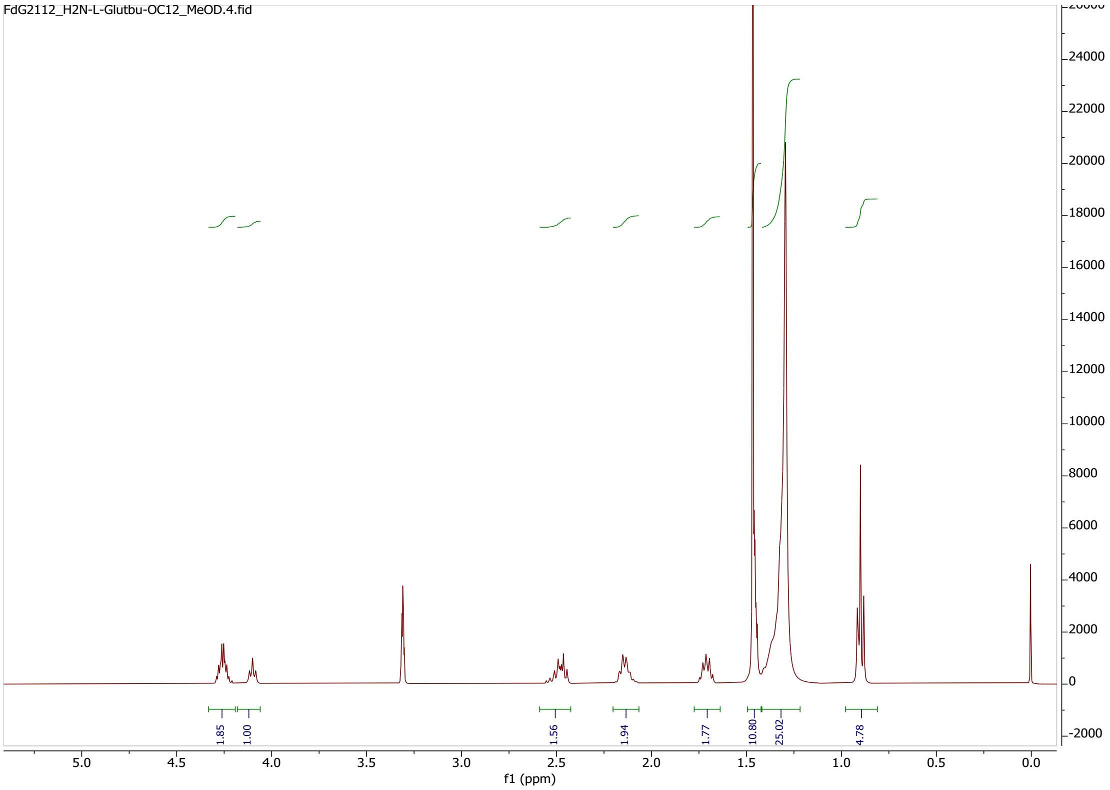
FdG2112_H2N-L-Glutbu-OC12_MeOD.4.fid
1.85
1.00
1.56
1.94
1.77
10.80
25.02
4.78
f1 (ppm)

(R)-2

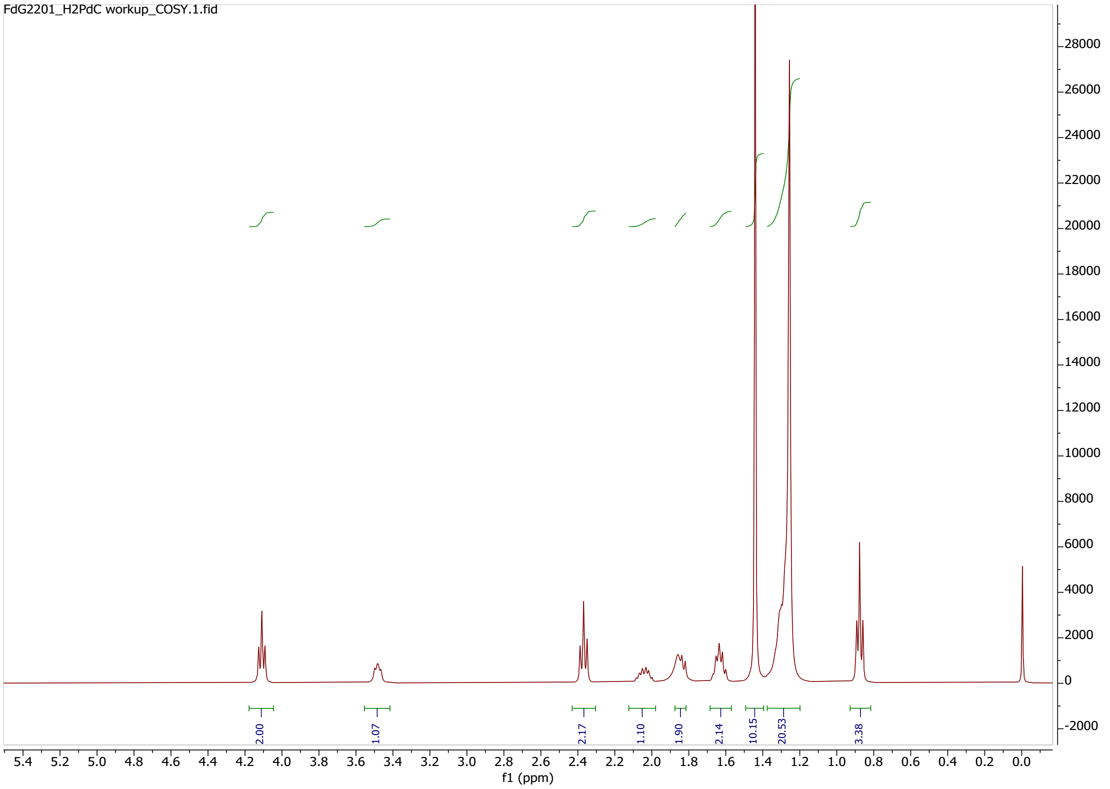
FdG2201_H2PdC workup_COSY.1.fid
2.00
1.07
2.17
1.10
1.90
2.14
10.15
20.53
3.38
f1 (ppm)

(S)-4

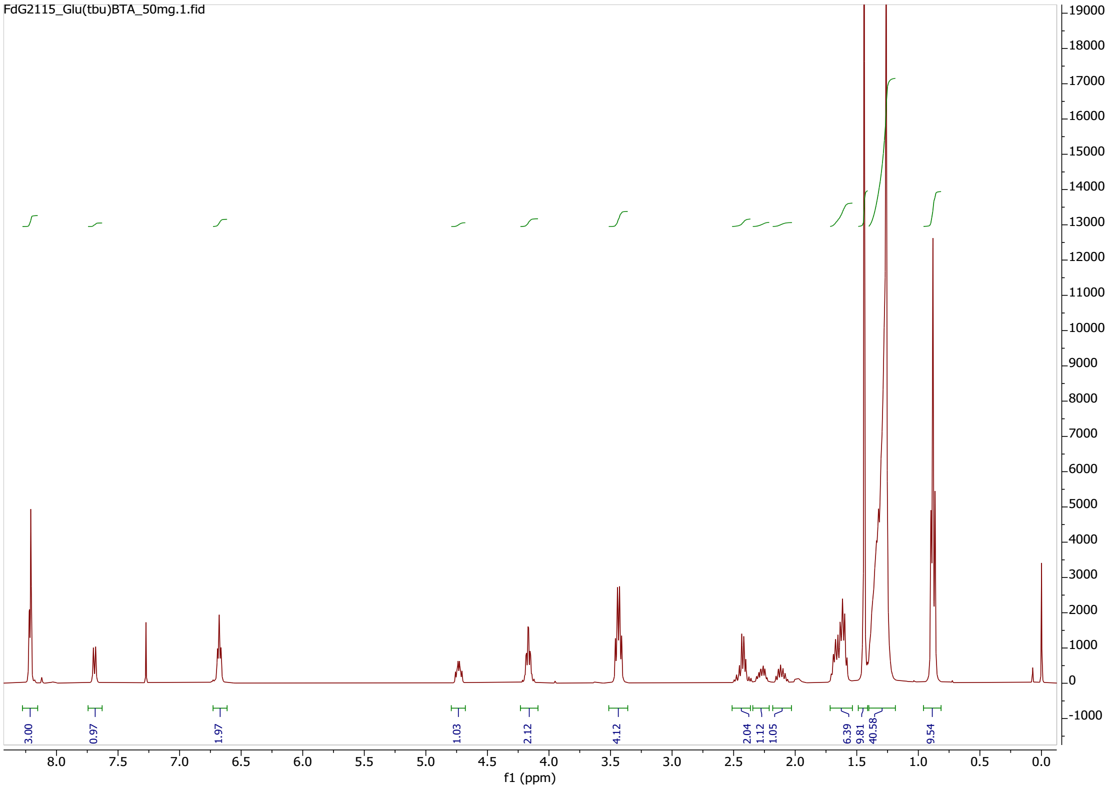
FdG2115_Glu(tbu)BTA_50mg.1.fid
3.00
0.97
1.97
1.03
2.12
4.12
2.04
1.12
1.05
6.39
9.81
40.58
9.54
f1 (ppm)

(R)-4

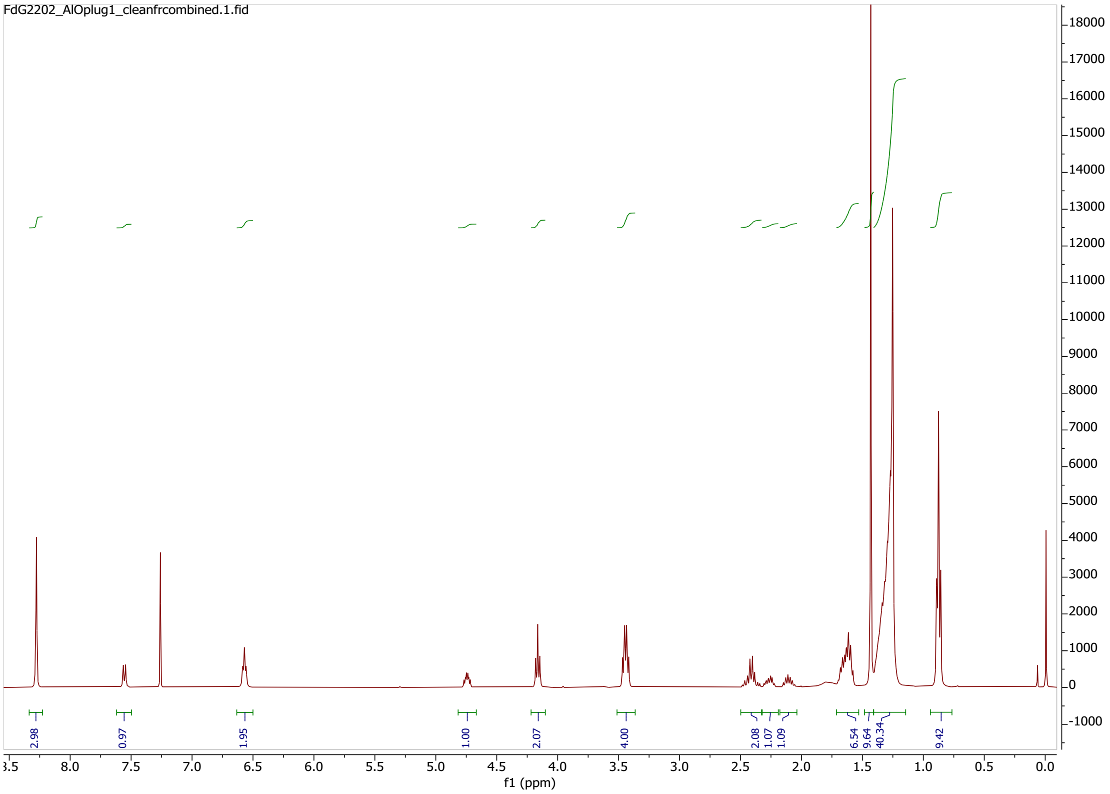
FdG2202_AlOplug1_cleanfrcombined.1.fid
2.98
0.97
1.95
1.00
2.07
4.00
2.08
1.07
1.09
6.54
9.64
40.34
9.42
f1 (ppm)

(S)-Glu(OMe)-BTA

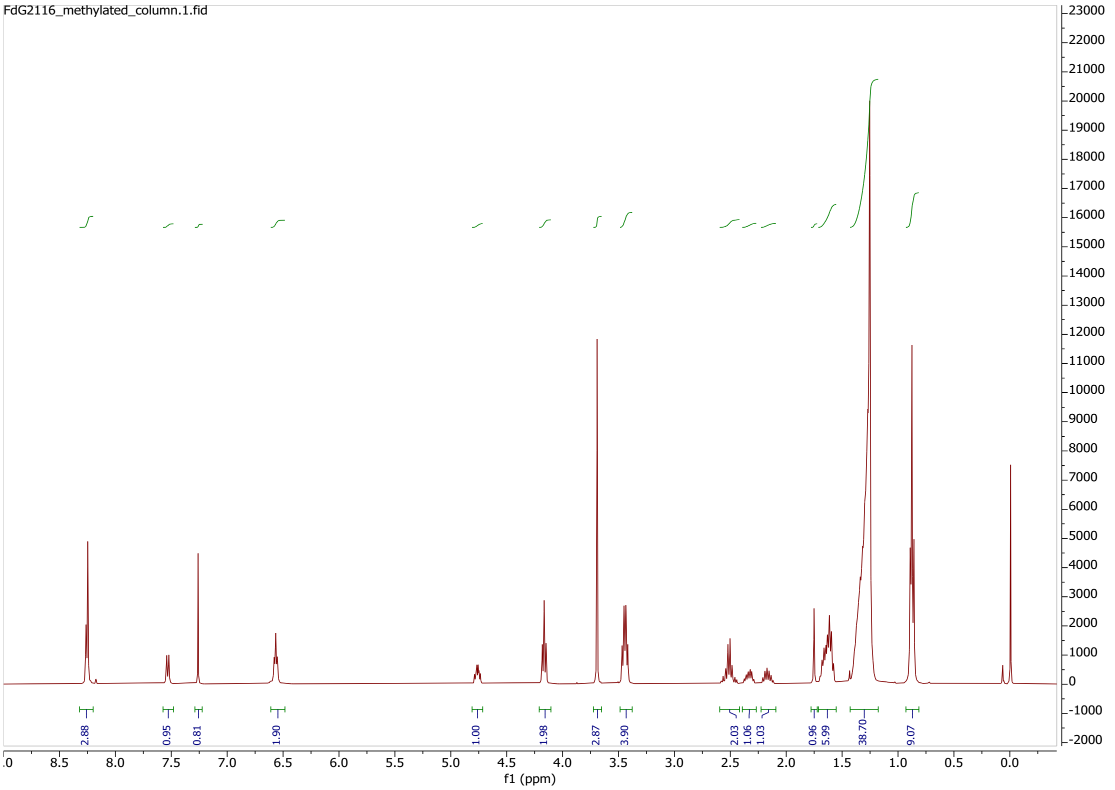

(R)-Glu(OMe)-BTA

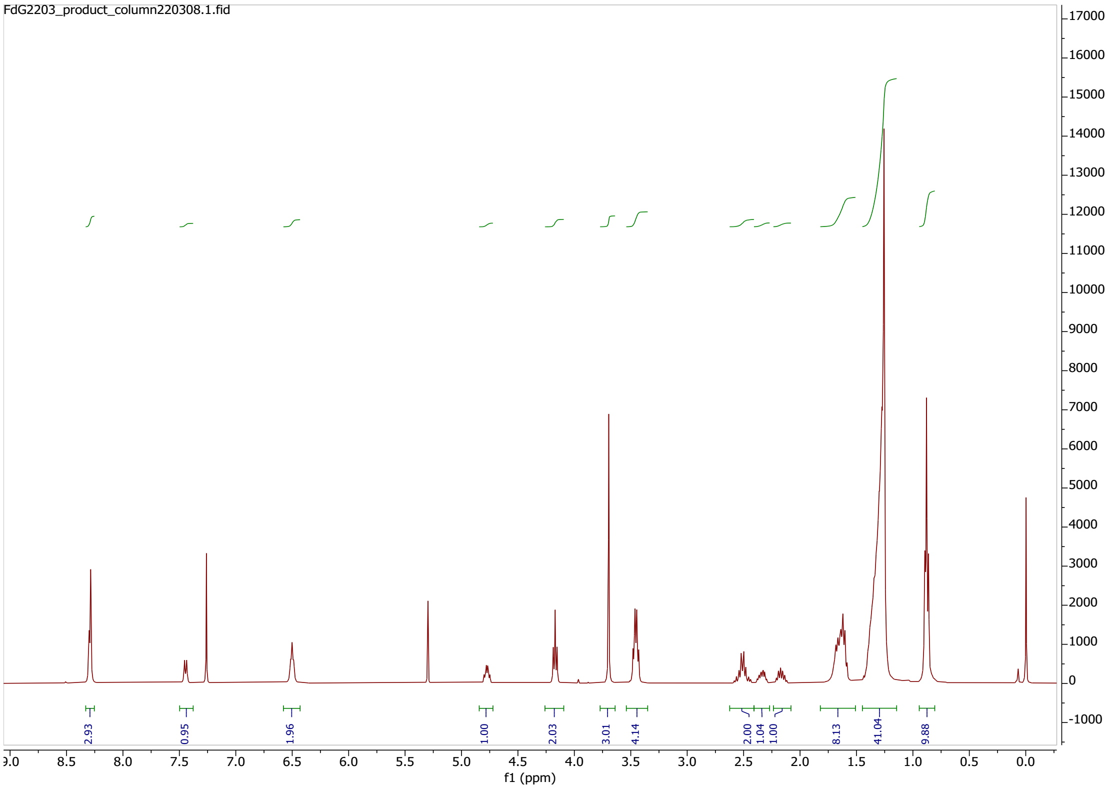

^{13}C NMRs

Compound 2

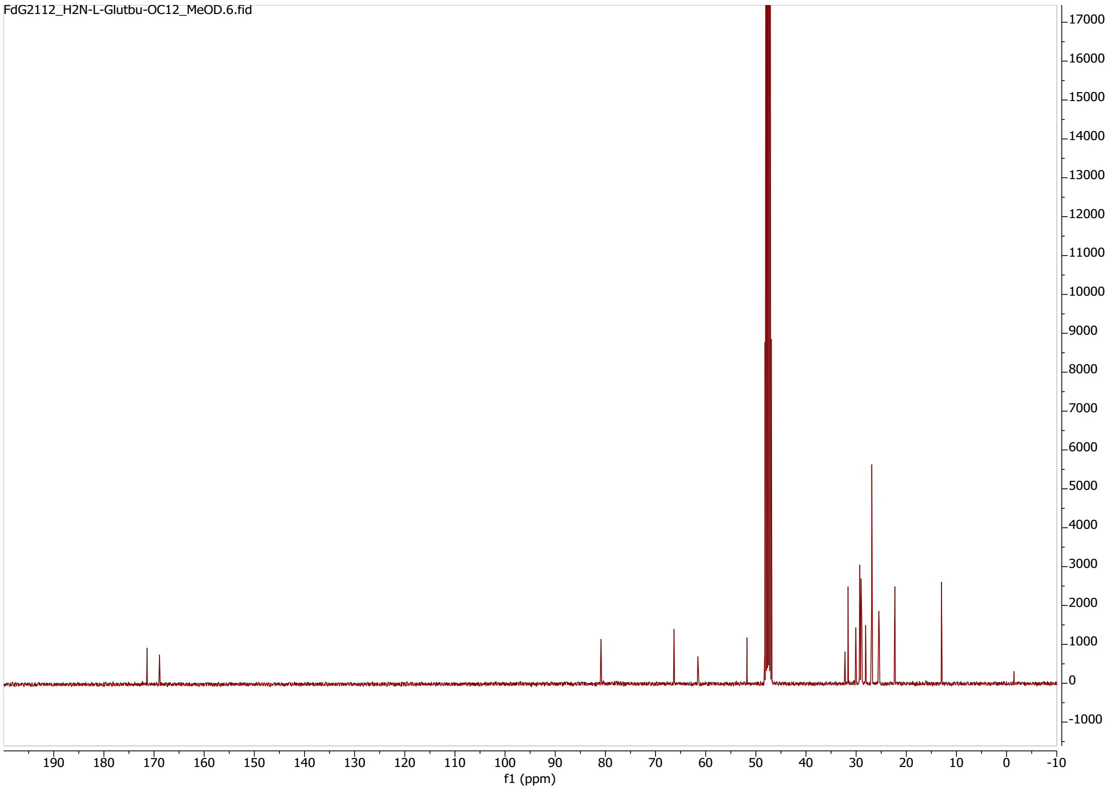

Compound 4

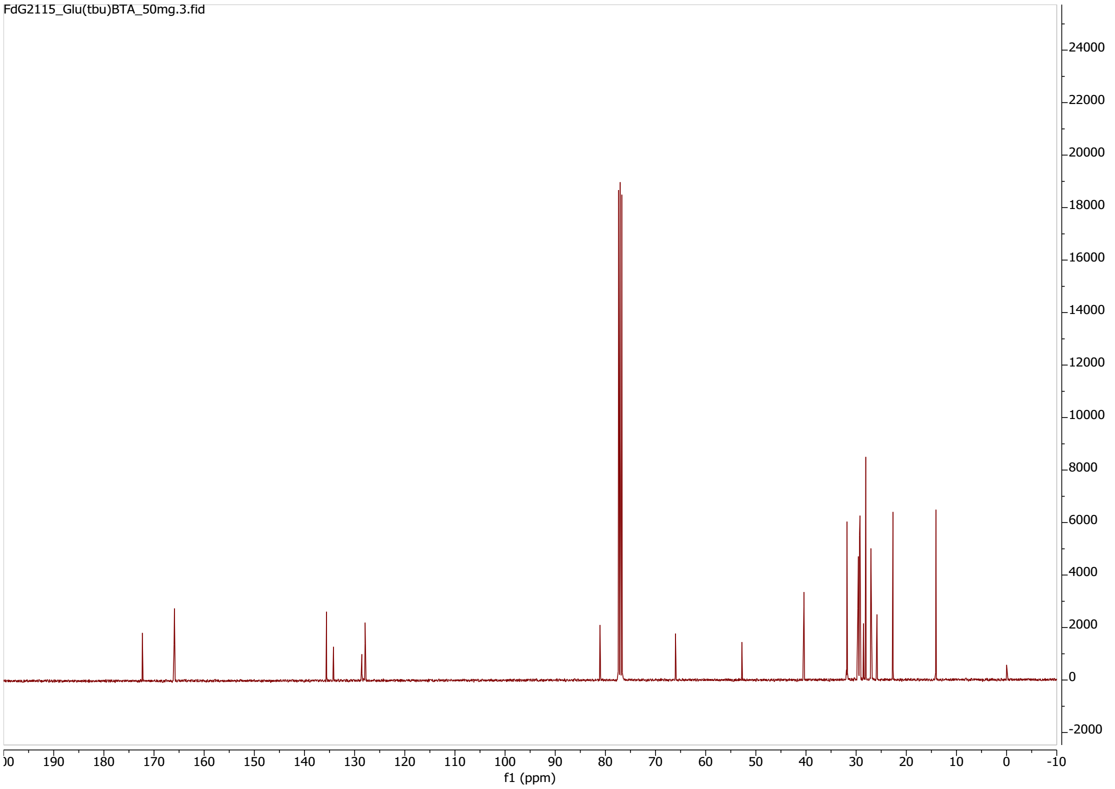

Glu-BTA

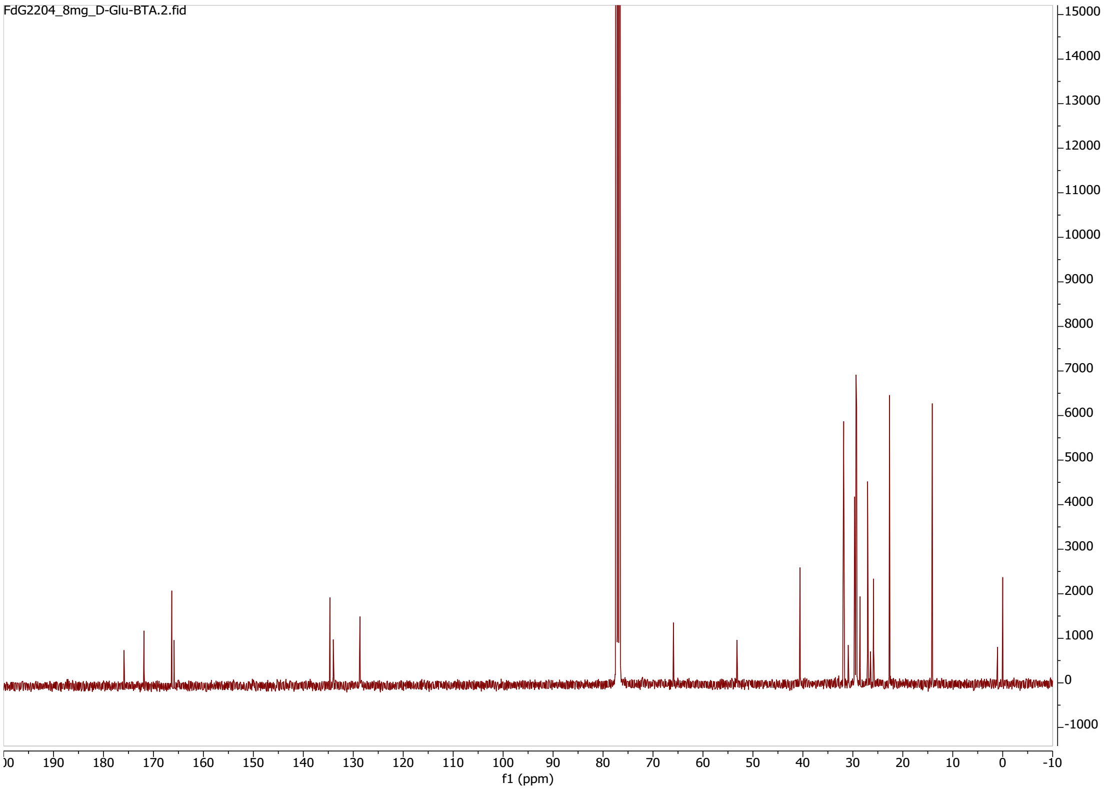

Glu(OMe)-BTA

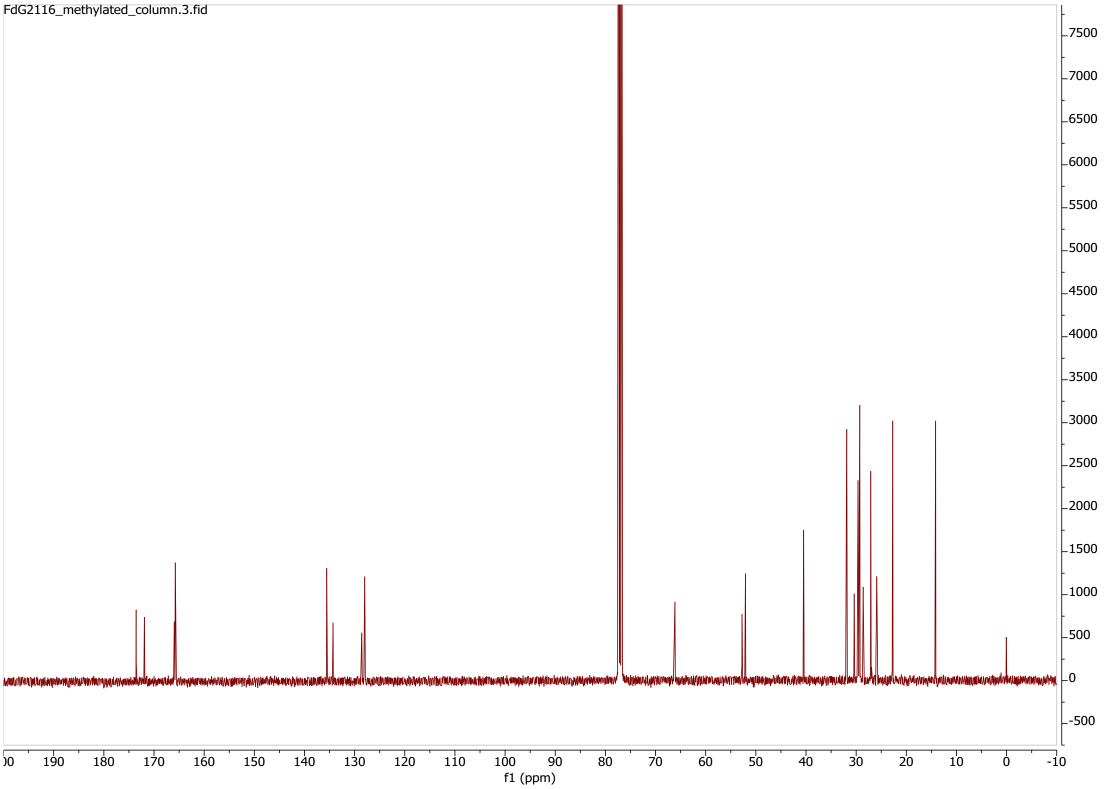

MALDI-TOF mass spectra

Compound 4 (contains traces of Glu-BTA)

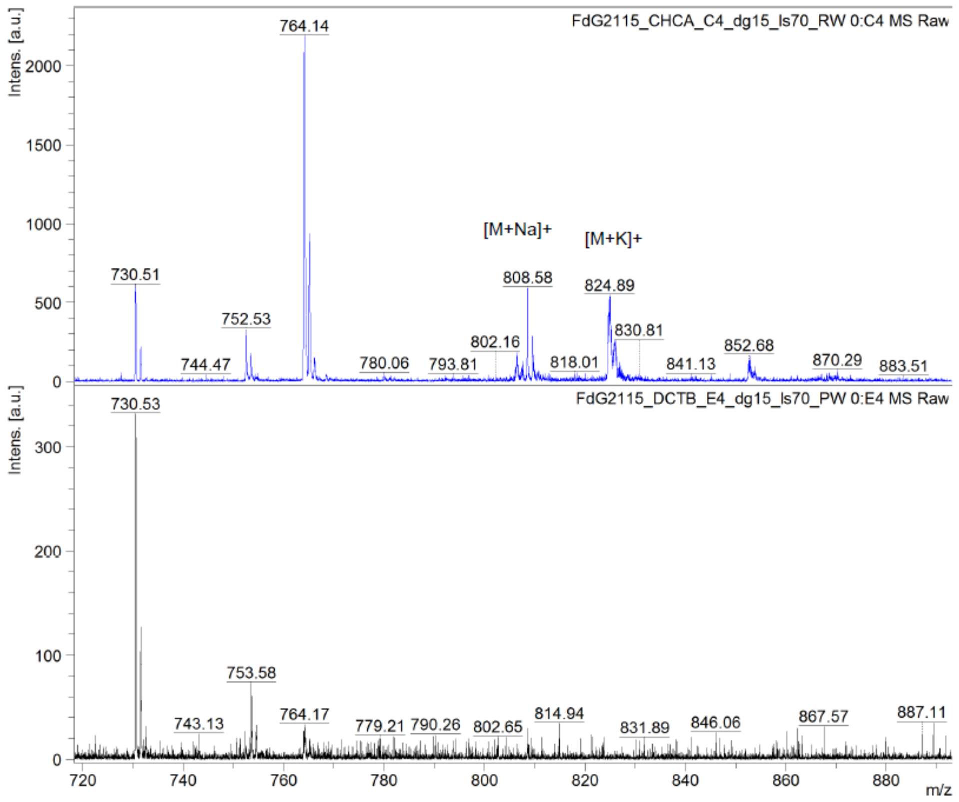

Glu-BTA

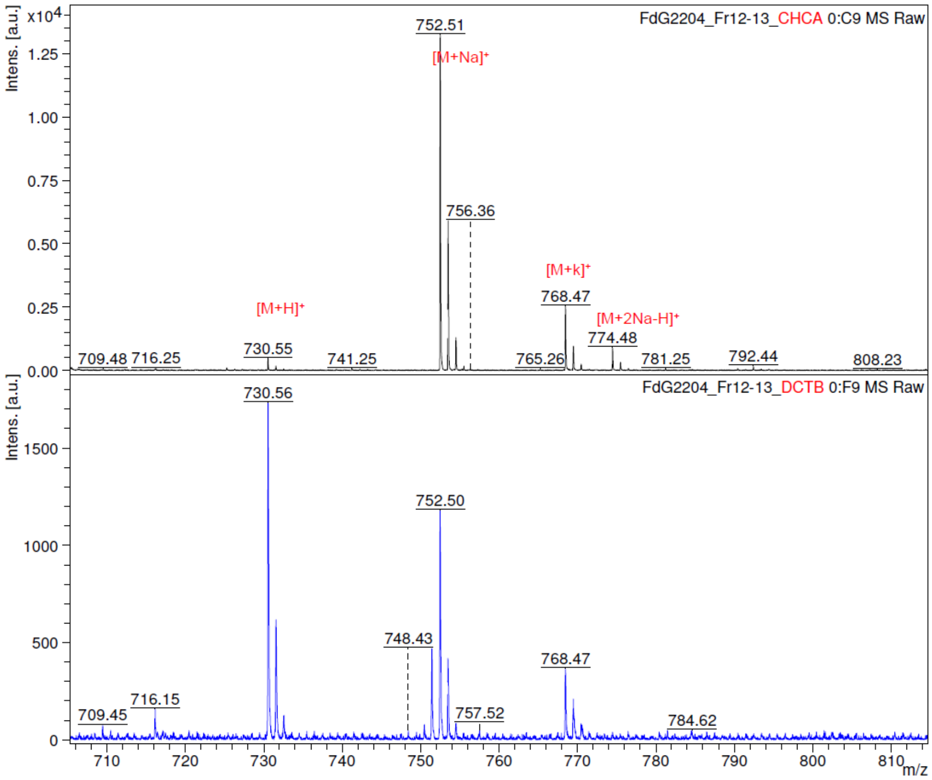

Glu(OMe)-BTA

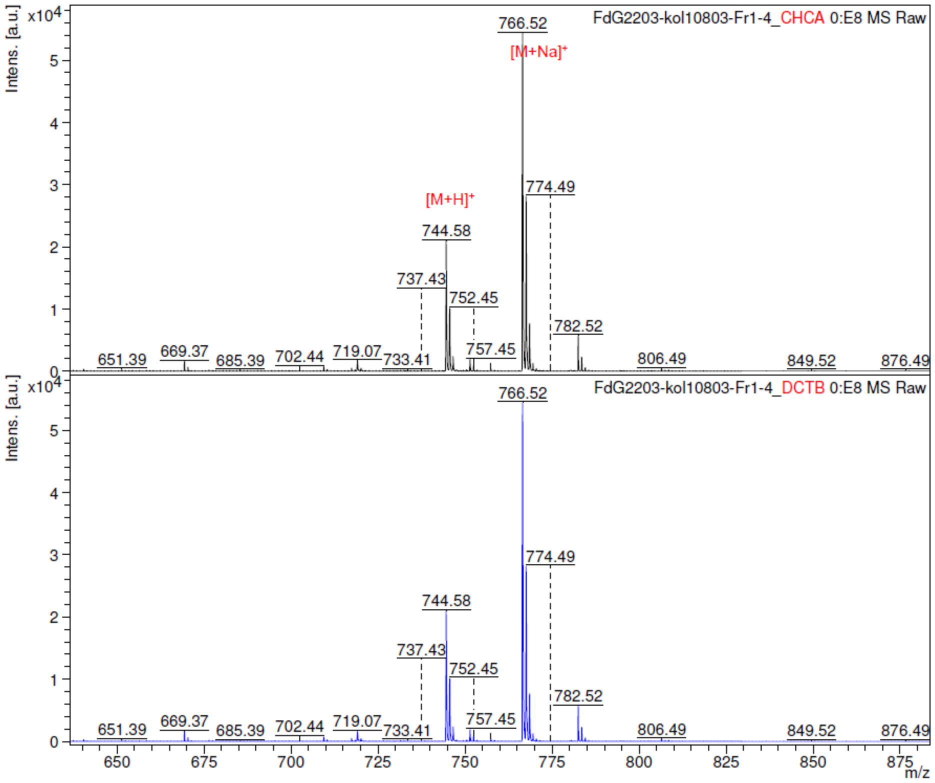

References

(1) Roosma, J.; Mes, T.; Leclère, P.; Palmans, A. R. A.; Meijer, E. W. Supramolecular Materials from Benzene-1,3,5-Tricarboxamide-Based Nanorods. *J. Am. Chem. Soc.* **2008**, *130* (4), 1120–1121.

(2) Zhao, D.; Moore, J. S. Nucleation-Elongation: A Mechanism for Cooperative Supramolecular Polymerization. *Org. Biomol. Chem.* **2003**, *1* (20), 3471–3491.

(3) Ten Eikelder, H. M. M.; Adelizzi, B.; Palmans, A. R. A.; Markvoort, A. J. Equilibrium Model for Supramolecular Copolymerizations. *J. Phys. Chem. B* **2019**, *123* (30), 6627–6642.

(4) Desmarchelier, A.; Alvarenga, B. G.; Caumes, X.; Dubreucq, L.; Troufflard, C.; Tessier, M.; Vanthuyne, N.; Idé, J.; Maistriaux, T.; Beljonne, D.; Brocorens, P.; Lazzaroni, R.; Raynal, M.; Bouteiller, L. Tuning the Nature and Stability of Self-Assemblies Formed by Ester Benzene 1,3,5-Tricarboxamides: The Crucial Role Played by the Substituents. *Soft Matter* **2016**, *12* (37), 7824–7838.

(5) Vonk, K. M.; Meijer, E. W.; Vantomme, G. Depolymerization of Supramolecular Polymers by a Covalent Reaction; Transforming an Intercalator into a Sequestrator. *Chem. Sci.* **2021**, *12*, 13572–13579.

(6) Presser, A.; Hüfner, A. Trimethylsilyldiazomethane - A Mild and Efficient Reagent for the Methylation of Carboxylic Acids and Alcohols in Natural Products. *Monatsh. Chem.* **2004**, *135* (8), 1015–1022.

(7) Kühnel, E.; Laffan, D. D. P.; Lloyd-Jones, G. C.; Martínez Del Campo, T.; Shepperson, I. R.; Slaughter, J. L. Mechanism of Methyl Esterification of Carboxylic Acids by Trimethylsilyldiazomethane. *Angew. Chem. Int. Ed.* **2007**, *46* (37), 7075–7078.
